# Platelets as crucial players in the dynamic interplay of inflammation, immunity, and cancer: unveiling new strategies for cancer prevention

**DOI:** 10.3389/fphar.2024.1520488

**Published:** 2024-12-23

**Authors:** Annalisa Contursi, Stefania Tacconelli, Sara Di Berardino, Alessandra De Michele, Paola Patrignani

**Affiliations:** ^1^ Systems Pharmacology and Translational Therapeutics Laboratory, The Center for Advanced Studies and Technology (CAST), “G. d’Annunzio” University, Chieti, Italy; ^2^ Department of Neuroscience, Imaging and Clinical Science, “G. d’Annunzio” University Medical School, Chieti, Italy

**Keywords:** platelets, cancer, aspirin, antiplatelet agents, inflammation, revacept, 12-lipoxygenase, extracellular vesicles

## Abstract

Inflammation plays a critical role in the pathogenesis of various diseases by promoting the acquisition of new functional traits by different cell types. Shared risk factors between cardiovascular disease and cancer, including smoking, obesity, diabetes, high-fat diet, low physical activity, and alcohol consumption, contribute to inflammation linked to platelet activation. Platelets contribute to an inflammatory state by activating various normal cells, such as fibroblasts, immune cells, and vascular cells. This activation is achieved by releasing diverse molecules from platelets, including lipids (eicosanoids), growth and angiogenic factors, and extracellular vesicles (EVs) rich in various RNA species. Antiplatelet agents like low-dose aspirin can prevent cardiovascular disease and cancer by inhibiting platelet functions beyond the antithrombotic action. Throughout the initial phases of tumorigenesis, the activation of platelets induces the overexpression of cyclooxygenase (COX)-2 in stromal cells, leading to increased biosynthesis of prostaglandin (PG)E_2_. This prostanoid can contribute to tumor development by inhibiting apoptosis, promoting cancer cell proliferation and migration, and immune evasion. Notably, platelets induce the epithelial-mesenchymal transition (EMT) in tumor cells, enhancing their metastatic potential. Two platelet eicosanoids, PGE_2_ (generated as a minor product of COX-1) and 12S-hydroxyeicosatetraenoic acid (HETE) [derived from the platelet-type 12-lipoxygenase (LOX)], contribute to EMT. In addition to the pharmacological inhibition of eicosanoid biosynthesis, a potential strategy for mitigating platelet-induced metastasis might encompass the inhibition of direct interactions between platelets and cancer cells. For example, there is promise in utilizing revacept to inhibit the interaction between platelet collagen receptors (particularly GPVI) and galectin-3 in cancer cells. Identifying these novel platelet functions suggests the potential application of antiplatelet agents, such as low-dose aspirin, in mitigating cancer risk, particularly in the case of colorectal cancer. It is necessary to investigate the effectiveness of other antiplatelet drugs, such as ADP P2Y_12_ receptor antagonists, in cancer prevention. Other new antiplatelet drugs, such as revacept and selective 12-LOX inhibitors, currently under clinical development, are of interest due to their low risk of bleeding. Platelets and EVs carry important clinical information because they contain specific proteins and RNAs associated with disease conditions. Their analysis can improve the accuracy of liquid biopsies for early cancer detection, monitoring progression, and assessing drug response.

## Introduction

### The cellular components involved in the process of inflammation

Acute inflammation is a highly regulated physiological process essential in defending the body against various external and internal threats. Any disruption in tissue homeostasis triggers the activation of innate immune cells, which form the first line of defense intended to restore the affected tissue (Delves and Roitt, 2000). The primary inflammatory cells that mediate acute inflammation are the polymorphonuclear leukocytes (PMN). The production of chemotactic molecules induces the migration from the venous system to the damaged site of monocytes/macrophages, mast cells, dendritic cells, and natural killer (NK) cells. They amplify the inflammatory response, leading to pathogen elimination and tissue repair by releasing cytokines, chemokines, matrix-remodeling proteases, and reactive oxygen and nitrogen species (Coussens and Werb, 2002; Chitu and Stanley, 2006). Like that associated with wound healing, physiological inflammation is a strictly controlled and self-limiting process (Martin and Leibovich, 2005). However, losing control over immune components can lead to chronic inflammation.

Multiple factors can contribute to an excessive burden of inflammation, encompassing lifestyle factors such as smoking and dietary habits, along with conditions like obesity, diabetes, and exposure to environmental pollutants (Libby, 2012). The development of chronic inflammation is associated with a range of diseases, including fatty liver disease, inflammatory bowel disease (IBD), Alzheimer’s, Parkinson’s, atherosclerosis, and cancer (Chen et al., 2017). Current studies indicate that the trigger signaling of chronic inflammation is associated with enhanced platelet activation (Gawaz et al., 2005; Dovizio et al., 2014). It is now acknowledged that platelets are mediators of intercellular communication and propagation of the cellular activation response through the release and transfer of their molecular cargo to numerous cell types implicated in inflammation (Rondina et al., 2013; Varon and Shai, 2015). Consequently, this insight opens doors to innovative approaches to curbing chronic inflammation-related diseases (Gawaz et al., 2023; Patrignani and Patrono, 2015; 2018).

In atherosclerosis, the damage of vascular endothelial cells induces a rapid adhering to the vascular wall of activated platelets; this event contributes to the development of vascular inflammation through leukocyte chemoattraction and their subsequent infiltration into the vessel wall associated with the proliferation of smooth muscle cells (Ross et al., 1985; Gawaz et al., 2005). Interactions between platelets and leukocytes occur via PSGL-1-P-selectin (Evangelista et al., 1999; Yang et al., 1999), followed by the binding between Mac-1 (CD11b/CD18, αMb2) and GPIbα (Simon et al., 2000), JAM-3 (Santoso et al., 2002), or ICAM-2 (Diacovo et al., 1994). The binding facilitates the release of inflammatory molecules from platelets, triggering monocyte inflammatory cascades (Weyrich et al., 1996).

Platelets promote plaque formation by accelerating foam cell formation upon binding to oxidized LDL (Coenen et al., 2021). Platelet-derived matrix metalloproteinases (MMPs), a family of proteolytic enzymes that mediate physiological and pathophysiological extracellular matrix turnover (Gresele et al., 2021), such as MMP-1 and -2 can cleave protease-activated receptors (PARs). Among them, PAR-1 is a G protein-coupled receptor with important roles in hemostasis and inflammation (Willis Fox and Preston, 2020). Rana et al. (2018) demonstrated a role for MMP-1 in plaque formation in mice, mediated via interaction with endothelial PAR-1. Momi et al. (2022) discovered that platelet MMP-2 plays a role in mice’s early formation of arterial plaque. This occurs by activating endothelial PAR-1, which leads to endothelial activation and monocyte intravasation. They also found that in patients with coronary artery disease (CAD) and human immunodeficiency virus (HIV), MMP-2 is overexpressed on the surface of platelets compared to healthy individuals of similar age and sex. Furthermore, it was noted that platelet MMP-2 levels are positively associated with the severity of carotid artery stenosis in humans. These findings indicate that inhibiting PAR-1 activities could be a promising way to reduce atherothrombosis. New strategies may involve targeting the signaling downstream of PAR-1 activated by MMP-1 and -2 to specifically inhibit the proinflammatory activity of PAR-1 (Willis Fox and Preston, 2020).

The release of platelet-derived CD40 ligand (CD40L, CD154) fosters inflammation within the endothelium. The binding of CD40 on endothelial cells to CD40L on activated platelets boosts the release of IL-8 and monocyte chemoattractant protein-1 (MCP-1), major attractants for neutrophils and monocytes (Henn et al., 1998). The interaction also prompts smooth muscle cells and macrophages to release MMPs, facilitating the degradation and remodeling of inflamed tissues.

A persistent inflammatory microenvironment continuously provides various factors—such as cytokines, chemokines, and growth factors—that oppose cell death and repair mechanisms, causing genomic instability and predisposing tissues to cancer (Mantovani and Pierotti, 2008; Hussain and Harris, 2007). Consequently, mediators and cellular effectors of inflammation play a crucial role in the local tumor milieu. It was estimated that 20% of all cancers are linked to chronic infections and inflammation (Mantovani et al., 2008), highlighting inflammation as the seventh hallmark of cancer. The unrestrained nature of cancer-related inflammation promotes tumor growth, supports angiogenesis and metastasis, undermines adaptive immune responses, and modifies responses to chemotherapy (Balkwill et al., 2005; Mantovani et al., 2008). Although genetic studies in mouse models have shown that innate immune cells can get an adaptive immune response capable of eradicating nascent tumors (Negus et al., 1997), the genetic, epigenetic, and metabolic instability characteristic of neoplastic cells allows new variants to escape immune surveillance, leading to tumor establishment and progression (immunoediting process) (Dunn et al., 2002).

Notably, leukocyte infiltration observed in tumor tissues and the anticancer efficacy of antiinflammatory agents support the role of chronic inflammation in cancer development and progression (Menter et al., 2010; Wong et al., 2020; Li et al., 2020).

**FIGURE 1:**
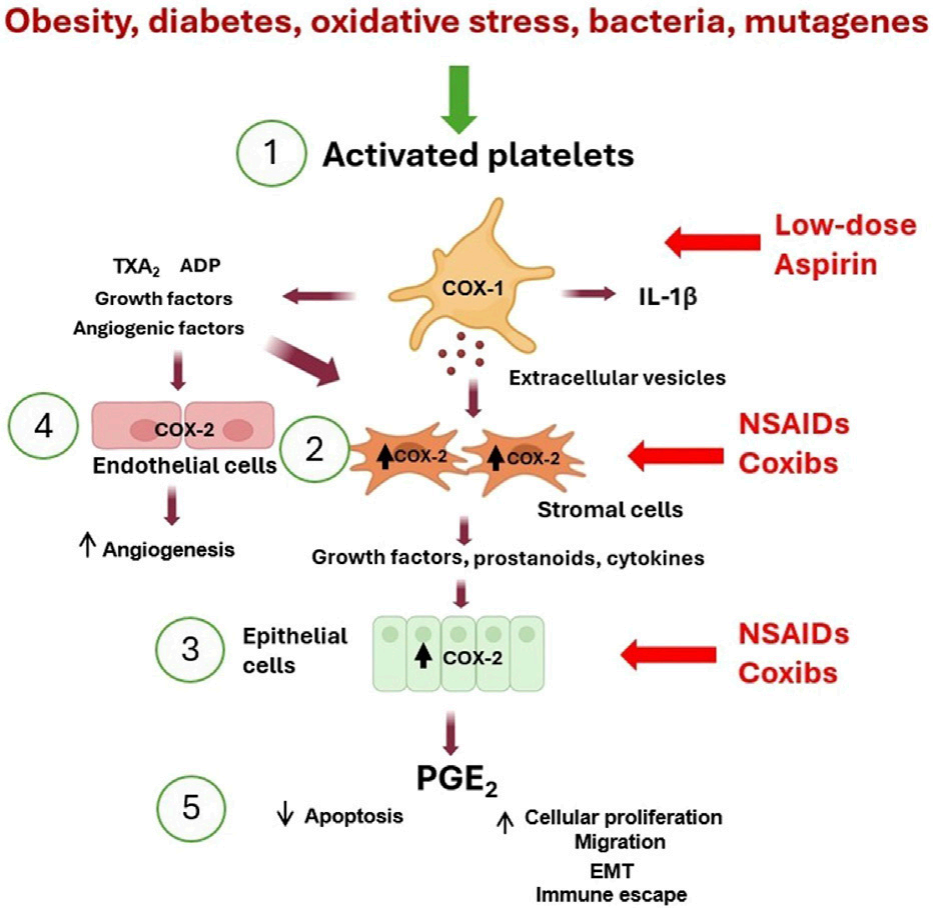
Platelet activation triggers the early phases of tumorigenesis. (1) Activated platelets release many mediators [including eicosanoids (such as TXA_2_), ADP, cytokines (such as IL-1β), growth and angiogenic factors, and extracellular vesicles (EVs) containing genetic material (such as mRNAs and microRNAs)] that contribute to (2) COX-2 overexpression in stromal cells (including macrophages and fibroblasts). (3) The crosstalk with epithelial cells leads to COX-2 induction. (4) Additionally, COX-2 is overexpressed in endothelial cells, leading to angiogenesis. (5) These events lead to the release of PGE_2_, which translates to inhibition of apoptosis, an increase in proliferation and migration, and induction of epithelial-mesenchymal transition (EMT). The inhibition of platelet COX-1 by low-dose aspirin prevents the downstream signaling pathways involved in the early events of tumorigenesis and indirectly inhibits COX-2 induction. In contrast, NSAIDs and coxibs have an antitumorigenic effect by directly inhibiting COX-2 activity and prostanoid generation in stromal cells, epithelial cells, and endothelial cells.

In the last decade, experimental evidence has supported platelets’ pivotal role in developing chronic inflammation beyond their roles in hemostasis and thrombosis (Davì and Patrono, 2007; Smyth et al., 2009; Patrignani and Patrono, 2015; 2018). Platelets transduce inflammation-related signals, contributing to the variable microenvironment facilitating cell plasticity and heterogeneity. Platelets interact directly with target cells and release numerous mediators, including eicosanoids [thromboxane (TX)A_2_ and prostaglandin (PG)E_2_], angiogenic and growth factors from α-granules, ADP from dense granules, and extracellular vesicles (EVs) containing genetic material such as mRNA and microRNAs (miRs) (Patrono et al., 2001; Patrignani and Patrono, 2015; 2018). These phenomena can contribute to developing the early events of tumorigenesis triggered by platelet activation (Figure 1).

### Platelet extravasation and the development of an inflammatory microenvironment

**FIGURE 2:**
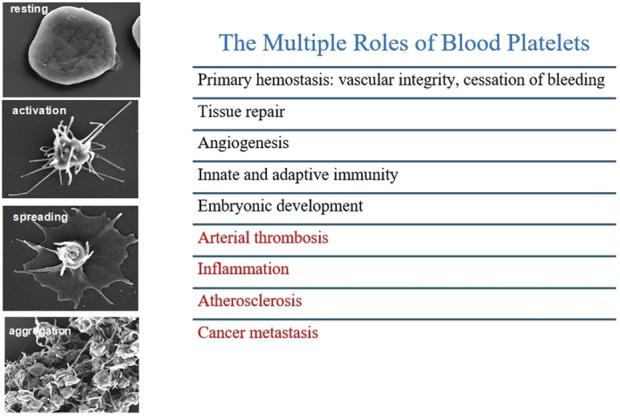
Different stages of platelet activation from resting to aggregation, by high resolution scanning electron microscopy. Platelets possess multifaceted functions beyond thrombosis contributing to inflammation, atherosclerosis and cancer metastasis.

Platelets possess multifaceted functions beyond thrombosis (Figure 2), significantly contributing to various pathological conditions, including atherosclerosis, restenosis, cardiac fibrosis, airway hyperresponsiveness, airway wall remodeling, intestinal colitis, and tumor metastasis (Patrignani and Patrono, 2018; Rumbaut and Thiyagaraja, 2010; Golebiewska and Poole, 2015). An important function of platelets is the capacity to extravasate at sites of inflammation, thus interacting and activating other cells that constitute the cellular stromal compartment of different tissues. Platelets activate other cell types via direct interaction or through the release of many mediators with a wide range of activities and the capacity to release EVs that transfer platelet cargo far from the platelet. These events are associated with inflammation, tissue injury, and organ failure severity.

Platelet recruitment to post-capillary venules at sites of acute inflammation has been shown in various experimental models, often associated with PMN-endothelial interactions. In a mouse model of corneal epithelial abrasion, an acute inflammatory response is necessary for effective wound healing (De La Cruz et al., 2021). In this model, PMNs and platelets are recruited to the small blood vessels surrounding the cornea in a mutually dependent manner. Depleting either cell type systemically inhibits the recruitment of the other. The mechanisms responsible for platelet extravasation in inflammation need to be clarified. However, the data suggest that in this model of corneal inflammation, platelet extravasation depends on CD18, mast cells, and PMNs, with a central role for mast cell degranulation in the responses. Platelet extravasation is accompanied by red blood cell (RBC) extravasation, with evidence of disruption of microvascular integrity (De La Cruz et al., 2021).

It has been reported that platelets influence leukocyte trafficking from blood vessels into lung tissue because platelets are necessary for the pulmonary recruitment of eosinophils and lymphocytes in murine allergic inflammation (Pitchford et al., 2003; Pitchford et al., 2005; Pitchford et al., 2008).

In hypertensive mice with prostacyclin (PGI_2_) receptor (IP) deletion (IPKO) fed with a high-salt diet associated with hypertension and cardiac fibrosis, enhanced systemic biosynthesis of TXA_2_ and left ventricular TXA_2_ receptor (TP) expression were detected (D’Agostino et al., 2021). Increased cardiac collagen deposition, profibrotic gene expression (including TGF-β), number of myofibroblasts at perivascular levels, and extravasated platelets were detected compared to WT mice treated with the same diet. The antiplatelet agent low-dose aspirin caused a selective inhibition of platelet TXA_2_ biosynthesis and mitigated enhanced blood pressure, cardiac fibrosis, and left ventricular profibrotic gene expression in IPKO but not WT mice. Moreover, the number of myofibroblasts and extravasated platelets in the heart was reduced (D’Agostino et al., 2021).

Platelets have been detected to extravasate and accumulate in the colonic lamina propria of chronic inflammation-associated fibrosis (Sacco et al., 2019) and intestinal adenomas in mice (Bruno et al., 2022), associated with an enhanced number of myofibroblasts.

### Platelet-myofibroblast crosstalk: a key mechanism of intestinal inflammation-linked tumorigenesis

Under normal physiological conditions, the stroma comprises fibroblasts, smooth muscle cells, immune cells, endothelial cells, nerve cells, and the extracellular matrix (ECM) (Barron and Rowley, 2012). The stroma is a structural and functional support system for epithelial cells, regulating their behavior and enabling tissue repair in response to injury (Tuxhorn et al., 2001). Stromal activation during wound healing, characterized by myofibroblast activation, type I collagen deposition, and angiogenesis, is also observed in the tumor microenvironment, referred to as reactive stroma (Kalluri and Zeisberg, 2006; Tuxhorn et al., 2002).

In intestinal inflammation resulting from epithelial damage, such as experimentally induced colitis in mice or inflammatory bowel disease (IBD) in humans, platelets are observed to migrate from the bloodstream into the interstitial tissue of the colon (Danese et al., 2004; Petito et al., 2017). This extravasation may contribute to the persistence of chronic inflammation, leading to fibrosis and facilitating tumorigenesis through intercellular communication with myofibroblasts.

Sacco et al. (2019) conducted a study revealing that when human platelets are cocultured with intestinal myofibroblasts, the platelets undergo activation, resulting in increased production of TXA_2_, a major oxylipin derived from arachidonic acid (AA) through cyclooxygenase (COX)-1 activity. Subsequently, TXA_2_ induces phenotypic and functional alterations in myofibroblasts. In the presence of platelets, the characteristic spindle-shaped myofibroblasts turn towards a polarized phenotype, accompanied by elongation of the cellular body associated with enhanced proliferation and migratory) properties. The changes in myofibroblast morphology and functions induced by platelets were accompanied by reduced α-SMA, vimentin, fibronectin, and RhoA expression. These changes were prevented when platelets were exposed to aspirin, an irreversible inhibitor of platelet COX-1, and then washed away before incubation with myofibroblasts. Similar effects were observed when using an antagonist of the TP receptors, indicating that COX-1-dependent TXA_2_ is a crucial mediator released by platelets to activate myofibroblasts via TP receptors (Sacco et al., 2019). In the coculture of human platelets and myofibroblasts, platelet-derived TXA_2_ was involved in the induction of COX-2 in myofibroblasts since it was prevented by the selective inhibition of platelet COX-1 by aspirin or by a specific antagonist of TXA_2_ receptors (TP) (SQ 29,548) (Bruno et al., 2022). Enhanced COX-2-dependent PGE_2_ was detected under these experimental conditions. Interestingly, both TXA_2_ and PGE_2_ contribute to COX-2 induction in the coculture of platelet and myofibroblasts. The activation of TP by the TXA_2_ mimetic U46619 caused a rapid expression of COX-2 in myofibroblasts cultured alone, but PGE_2_ was required for the sustained expression of COX-2 (Bruno et al., 2022). Faour et al. (2001) reported that in IL-1β-treated human synovial fibroblasts, PGE_2_ stabilizes COX-2 mRNA and stimulates translation via EP4 receptor and the downstream kinases p38MAPK and cAMP-dependent protein kinase.

Enhanced COX-2-dependent PGE_2_ in the tumor microenvironment promotes tumor growth and inhibition of immunosurveillance and induces angiogenesis. Moreover, PGE_2_ can activate tumor epithelial cells, causing proliferation, survival, migration/invasion, and epigenetic changes (Wang and DuBois, 2013). However, it was found that TXA_2_ and PGE_2_ contribute to COX-2 induction in the coculture of platelet and myofibroblasts. The activation of TP by the TXA_2_ mimetic U46619 caused a rapid expression of COX-2 in myofibroblasts cultured alone, but PGE_2_ was required for the sustained expression of COX-2 (Bruno et al., 2022). Faour et al. (2001) reported that in IL-1β-treated human synovial fibroblasts, PGE_2_ stabilizes COX-2 mRNA and stimulates translation via EP4 receptor and the downstream kinases p38MAPK and cAMP-dependent protein kinase.

Proinflammatory stimuli, such as IL-1β, have been shown to upregulate COX-2 signaling in human colonic fibroblasts, modulating the proliferation and invasiveness of human colonic epithelial cancer cells (Zhu et al., 2012). Moreover, COX-2 expression has been detected in the stromal compartment of polyps from *Apc* ^*Min/+*^ mice (Oshima et al., 1996).

These findings demonstrate the potential contribution of platelets to the development of an inflammatory microenvironment, thereby promoting the early stages of tumorigenesis through the upregulation of COX-2 and the increased production of PGE_2_ (Figure 1). Platelet-derived TXA_2_ is a pivotal trigger and sustainer of the cascade of events induced by platelet activation leading to intestinal tumorigenesis. Notably, antiplatelet agents can indirectly disrupt this cascade of downstream events (Patrignani and Patrono, 2015). Direct inhibition of COX-2 activity can achieve similar efficacy. Selective COX-2 inhibitors (coxibs) reduce tumor development by targeting COX-2 and PGE_2_. (Bertagnolli et al., 2006; Patrignani and Patrono, 2015; Wang and DuBois, 2013) (Figure 1). However, the heightened risk of cardiovascular side effects by COX-2 inhibitors hinders the utilization of these drugs for long-term cancer prevention regimens (Grosser et al., 2006).

### The role of platelets in the development of intestinal tumorigenesis: a lesson from conditional COX-1 knockout mice in megakaryocytes/platelets

The role of platelet TXA_2_ in intestinal inflammation and tumorigenesis has been convincingly demonstrated by generating mice with the specific deletion of COX-1 in megakaryocytes/platelets (Sacco et al., 2019; Bruno et al., 2022).

We generated a mouse with floxed Ptgs1 (COX-1) (Sacco et al., 2019), in which exons 6 and 7 were flanked by loxP sites using transcription activator-like effector nucleases as a genome-editing tool, which significantly boosts genomic modification efficiency (Joung and Sander, 2013). These mice were bred with platelet factor 4 (Pf4)-Cre transgenic C57BL/6 mice [obtained from Jackson Laboratories (Bar Harbor, ME)] expressing a codon-improved Cre recombinase (iCre) under the control of the mouse Pf4 promoter, resulting in Cre recombinase expression in most megakaryocytes (Sacco et al., 2019). In these mice, the synthesis of TXA_2_ by platelet COX-1 was almost completely inhibited. The systemic biosynthesis of TXA_2_, as assessed by the enzymatic urinary metabolites of TXB_2_, was also profoundly reduced. However, COX-2-dependent markers of prostanoid biosynthesis (such as the urinary metabolite of PGE_2_ and PGI_2_) were not significantly affected (Sacco et al., 2019; Bruno et al., 2022). These effects observed in mice with the COX-1 specific deletion in platelets are similar to the impact of low-dose aspirin in humans, which is an antiplatelet agent that selectively inhibits COX-1 in platelets (Patrignani et al., 2014; Patrono et al., 2005).

In mice with experimental intestinal colitis treated with dextran sulfate sodium (DSS), there is increased biosynthesis of TXA_2_ from platelets (Sacco et al., 2019). TXA_2_ contributes to increased microvascular permeability (Turnage et al., 1985) and inflammatory, immune cells, and platelets’ movement, leading to colon accumulation (Vitiello et al., 2014). Deletion of COX-1 in platelets inhibits the chronic inflammatory response linked to the reversal of intestinal colitis symptoms in DSS-induced colitis. Furthermore, the specific deletion of COX-1 in platelets prevented intestinal inflammation-associated fibrosis (Sacco et al., 2019).

**FIGURE 3:**
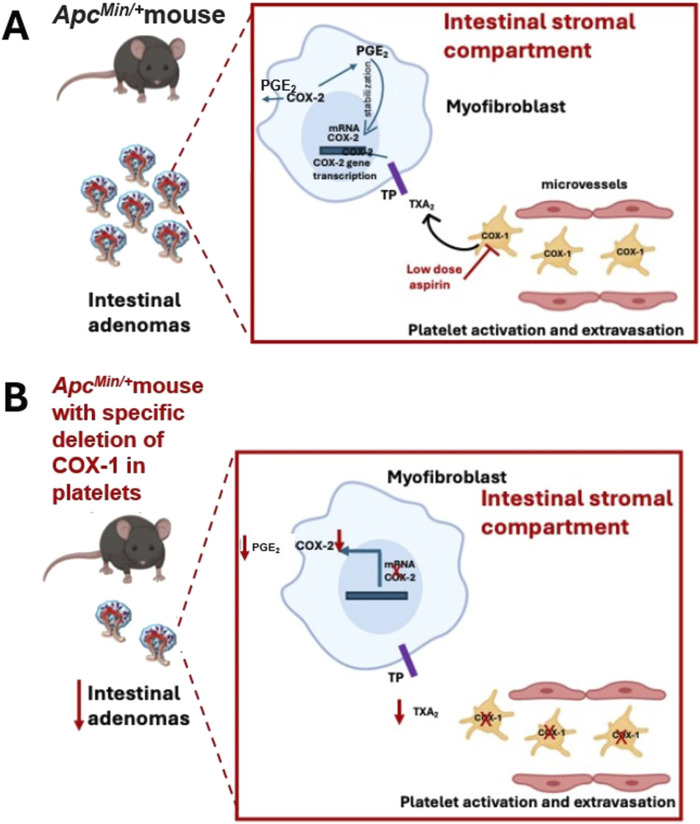
The role of platelet COX-1 in intestinal tumorigenesis of *Apc* ^*Min/+*^ mice. **(A)** The platelet COX-1-dependent TXA_2_ (also from extravasated platelets) can contribute to intestinal neoplasia by triggering the expression of COX-2 in stromal cells, which is involved in the biosynthesis of PGE_2_; the selective inhibition of COX-1 in platelets by aspirin can prevent the biosynthesis of TXA_2_ and indirectly the upregulation of COX-2. **(B)** In *Apc* ^*Min/+*^ mice, the specific deletion of platelet COX-1 caused a profound reduction in platelet TXA_2_ biosynthesis *in vivo*, which was associated with decreased COX-2 expression and PGE_2_ biosynthesis, and a reduced number and size of intestinal adenomas.

In *Apc* ^*Min/+*^ mice that develop multiple intestinal neoplasia due to a nonsense mutation at codon 850 of the *Apc* gene, like humans with germline mutations in the *APC* gene (Moser et al., 1995), enhanced systemic TXA_2_ biosynthesis was detected deriving mainly from activated platelets (Bruno et al., 2022). The specific deletion of platelet COX-1 in *Apc* ^*Min/+*^ mice was associated with a reduced systemic biosynthesis of TXA_2_ and a reduced number of intestinal adenomas (Figures 3A, B). *Apc* ^*Min/+*^mice developed multiple sessile tubular adenomas with marked nuclear enlargement with hyperchromasia, denoting a lower grade of differentiation than *Apc* ^*Min/+*^ mice with the specific deletion of platelet COX-1, as confirmed by the higher number of mitotic elements (Bruno et al., 2022). The adenomas of *Apc* ^*Min/+*^mice showed higher staining of PCNA (proliferating cell nuclear antigen) than *Apc* ^*Min/+*^mice with specific deletion of COX-1 in the platelet; PCNA is an auxiliary protein for DNA polymerase that reaches maximal expression during the S phase of the cell cycle (Celis et al., 1987). In polyps of *Apc* ^*Min/+*^mice, enhanced expression of COX-2 and mPGES-1 (i.e., microsomal prostaglandin E synthase-1, the downstream enzyme responsible for PGE_2_ biosynthesis from COX-2 product PGH_2_) was detected in association with the downregulation of 15-prostaglandin dehydrogenase (15-PGDH, an enzyme oxidizing and degrading PGE_2_). These changes could contribute to enhanced biosynthesis of PGE_2_ in the tumor, which can reflect the increased systemic biosynthesis of PGE_2_ found in *Apc* ^*Min/+*^ mice (Bruno et al., 2022). Deleting platelet COX-1 prevented the upregulation of COX-2 in the intestinal adenomas associated with reduced systemic biosynthesis of PGE_2_ (Figure 3B). The results of this study demonstrate that activated platelets can contribute to enhanced PGE_2_ generation in intestinal tumors, and inhibition of platelet function can indirectly prevent it, thus interfering with adenoma formation (Figures 3A, B).

### Platelets and the immune system

It is increasingly evident that platelets play a pivotal role in various immunological processes beyond their conventional function in hemostasis and thrombosis (Ali et al., 2015). Platelets can contribute to 1) defending against microbial threats, 2) recruiting and augmenting innate effector cell functions, 3) regulating antigen presentation, and 4) enhancing adaptive immune responses.

**FIGURE 4:**
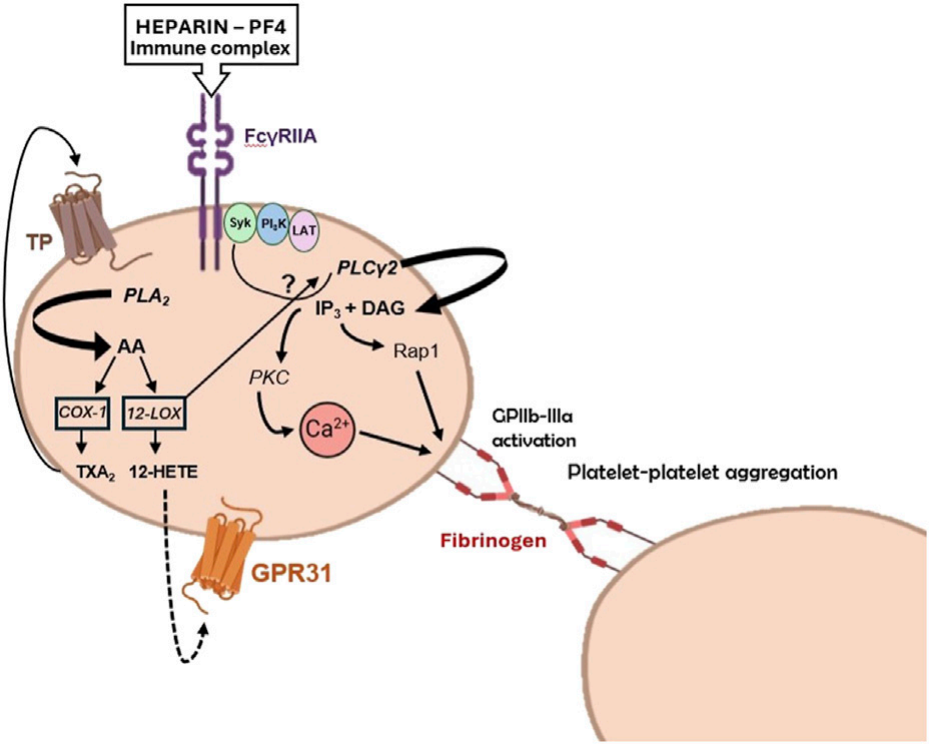
Potential roles of 12-lipoxygenase (LOX) in platelet activation by heparin-platelet factor 4 (PF4) immune complexes. PF4 is a chemokine released from platelet a-granules upon activation. It can form immune complexes with negatively charged substances, such as heparin. The formation of PF4-heparin complexes leads to the synthesis of antibodies, which activate platelets via FcγRIIa receptors. 12-LOX can amplify platelet activation through FcγRIIa via three different pathways: i) the arachidonic acid (AA)-derived compound 12S-HETE generated by 12-LOX, ii) the 12S-HETE receptor GPR31, and iii) the interaction with PLCγ2 or upstream signaling and adaptor molecules, such as Syk (spleen tyrosine kinase), PI3K (phosphoinositide 3-kinase), and LAT (T-cell activation linker). Modified from Alison H. Goodal et al. Blood 2014; 124 (14): 2166–2168 (comment on Yeung et al., Blood 2014; 124:2271-2279).

Platelets interact with immune complexes (ICs), which are associations of antibodies with their antigens. The presence of ICs in the bloodstream is documented in many pathological conditions characterized by chronic or acute inflammation (Cloutier et al., 2018). Platelets become activated when ICs bind to the IgG Fc receptor on human platelets, known as FcγRIIa (CD32) (Arman and Krauel, 2015). FcγRIIa is one of the three immunoreceptor-based activation motif (ITAM) family members of tyrosine kinase signaling receptors in human platelets (Boylan et al., 2008). The other two members are the collagen receptor glycoprotein (GP) VI and C-type lectin receptor 2. These receptors have a different signaling pathway from the G protein-coupled receptors (GPCRs) for other platelet agonists such as thrombin, adenosine 5′-diphosphate (ADP), and TXA_2_ (Boulaftali et al., 2014). Upon activation via ITAMs, platelets undergo aggregation and degranulation and are transformed into procoagulant platelets, which release procoagulant EVs (Puhm et al., 2021). Platelets express COX-1, crucial in converting AA released from membrane phospholipids following activation to the platelet agonist TXA_2_ (Patrignani and Patrono, 2015; 2018) that activates platelets through their TP receptors (Rovati et al., 2022). The selective and irreversible inhibition of COX-1 by aspirin is a fundamental aspect of antiplatelet therapy (Patrignani and Patrono, 2015). However, platelets also express 12-lipoxygenase (12S-LOX) (Contursi et al., 2022; Yoshimoto et al., 1992), and its specific role in platelet biology is not fully elucidated. AA released from cellular phospholipids in platelets can undergo a metabolic pathway involving 12-LOX (Figure 4), resulting in the generation of 12S-HpETE (hydroperoxyeicosatetraenoic acid), which subsequently undergoes rapid conversion to 12S-HETE (hydroxyeicosatetraenoic acid). The functional implications of 12-HETE in platelet physiology were hindered by the lack of specific 12-LOX inhibitors (Contursi et al., 2022). However, inhibitors that are selective for 12S-LOX over other LOXs and COXs have recently been developed (Luci et al., 2014). Their use showed the involvement of 12-LOX in the aggregation and degranulation of platelets activated by collagen, ADP, and thrombin, the latter via the PAR4 receptor, but interestingly, not via PAR1 (Tourdot and Holinstat, 2017). Using one of these new 12-LOX inhibitors (ML355), it was evidenced that 12-LOX also plays an important role in activating platelets through FcγRIIa (Yeung et al., 2014; Luci et al., 2014).

Heparin-induced thrombocytopenia (HIT) is a condition where platelet count decreases due to the disintegration and release of platelet-derived EVs into the bloodstream, promoting thrombin generation (Ahmed et al., 2007). It can be life-threatening and is caused by heparin binding to platelet factor 4 (PF4) released from platelets, which in some patients can result in immune recognition of the structurally modified heparin-PF4 complexes (Arepally and Padmanabhan, 2021). In HIT, 12-LOX is important in stimulating platelets through FcγRIIa leading to GPIIb-IIIa (the platelet fibrinogen receptor) activation (Goodall, 2014) (Figure 4). Thus, ML355 attenuates aggregation and degranulation via a mechanism that also affects phosphorylation of phospholipase Cγ2 (PLCγ2), calcium mobilization, and phosphorylation of protein kinase C (PKC) and Ras-proximate-1 or Ras-related protein 1 (Rap1involved in activation of GPIIb-IIIa) (Figure 4), while having no direct effect on phosphorylation of FcγRIIa itself (Yeung et al., 2014). The decreased phosphorylation of Rap1 was confirmed in platelets from 12-LOX^−/−^ mice, indicating that the effects were specific to 12-LOX. The data suggests that targeting 12-LOX could be a promising treatment for patients with HIT. Preclinical studies have shown that ML355 has a good safety profile (Adili et al., 2017). As 12-LOX enhances platelet activation by other agonists like TXA_2_ and ADP, selectively inhibiting 12-LOX may reduce the platelet response without completely blocking it. This indicates a favorable balance between the risk of clotting and bleeding. ML355 (also known as VLX-1005) is currently in phase 2 clinical trials for treating HIT and thrombosis (Stanger and Holinstat, 2023).

Due to immune surveillance, tumor cells face significant challenges surviving in the bloodstream. Consequently, metastasis and subsequent extravasation involve intricate mechanisms facilitating cancer cell survival in the systemic circulation. Fundamental to this process is the recruitment of monocytes, neutrophils, and platelets, which protect tumor cells from immune surveillance and facilitate the formation of metastases (Kitamura et al., 2015). In addition to these mechanisms, platelets have been found to impair the immune response through various other avenues. They play a pivotal role in regulating innate and adaptive immunity, particularly T cells. Furthermore, platelets can serve as antigen-presenting cells (APCs), as demonstrated by the expression of MHC class I proteins on their surface, initiating the adaptive immune response (Ali et al., 2015). It has been reported that platelets can transfer MHC class I proteins to tumor cells, resulting in a tumor cell phenotype termed the “phenotype of false pretenses.” This allows platelets to disrupt the self from non-self-recognition by NK cells, ultimately failing to protect the host by producing IFN-γ (Placke et al., 2012).

**FIGURE 5:**
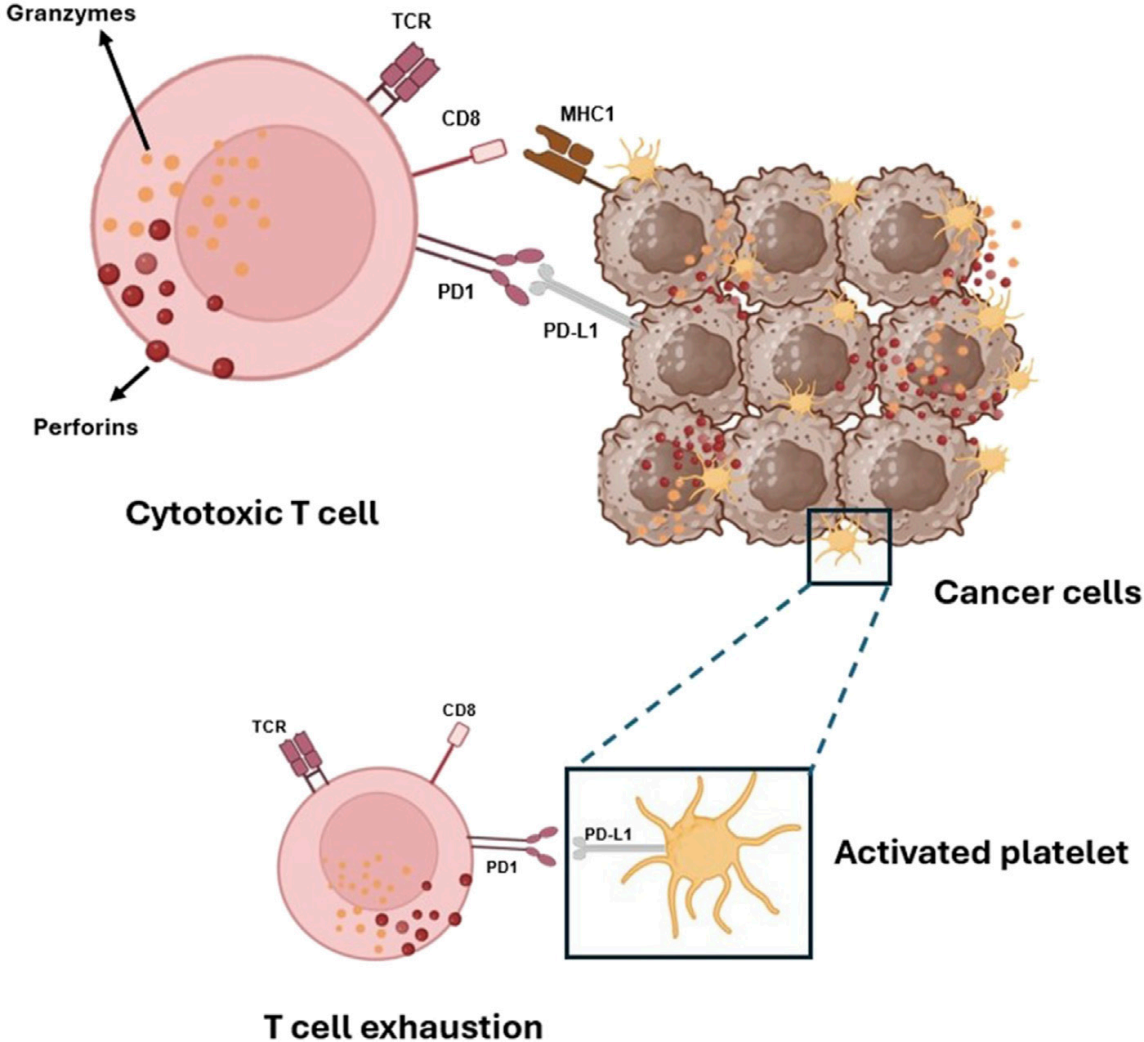
Platelets and tumor cells’ immune evasion via activation of PD-1 on T-cells by its ligands PD-L1. Platelets, like cancer cells, can activate the immune checkpoint protein PD-1, which controls the immune response of cytotoxic CD^8+^ T cells that carry out their killing function by releasing two types of preformed cytotoxic proteins: the granzymes, which seem able to induce apoptosis in any target cell, and the pore-forming protein perforin, which punches holes in the target cell membrane through which the granzymes can enter.

Emerging evidence indicates that platelets express the immune checkpoint molecule PD-L1 (Programmed Death-Ligand 1), which interacts with PD-1 (Programmed Cell Death Protein 1) on T cells, leading to their exhaustion (Figure 5). Notably, platelets from cancer patients with non-small cell lung cancer (NSCLC) showed significant levels of PD-L1 (Hinterleitner et al., 2021). Using specific antibodies, such as pembrolizumab, to block the PD-L1/PD-1 interaction has resulted in long-term responses in patients with metastatic NSCLC (Dang et al., 2016). Some tumor cells also express high levels of PD-L1, aiding in immune system evasion (Juneja et al., 2017).

Interestingly, Rachidi and colleagues (2017) identified a potential role for the non-signaling TGF-β-docking receptor glycoprotein A repetitions predominant (GARP), constitutively expressed on the platelet surface. Platelets are the primary source of TGF-β in systemic circulation and the tumor microenvironment. Platelet TGF-β production and secretion occur through GARP expression and the secretion of TGF-β itself (Tran et al., 2009). It has been shown that platelet-derived TGF-β has an immunosuppressive effect, primarily affecting T cells, and that the deletion of Lrrc32 (the gene encoding GARP) enhances the protective effect of the immune system against both melanoma and colon cancer. Combining immunotherapy with antiplatelet agents is an effective therapeutic strategy in mouse models. This effectiveness is attributed to the interruption of the GARP-TGF-β axis (Rachidi et al., 2017). Thus, antiplatelet agents may interfere with cancer cells’ capacity to evade immune surveillance (Patrignani and Patrono, 2018).

Platelets appear to be involved in the pathogenesis of hepatocellular carcinoma (HCC), a potential complication of chronic HBV infection (Sitia et al., 2012). During chronic hepatitis B virus (HBV) infection, HBV-specific CD8^+^ T cells fail to eradicate the infection, leading to necrosis, regeneration, inflammation, and ultimately HCC development (Guidotti and Chisari, 2006). It was reported that administering aspirin and clopidogrel (two antiplatelet agents) reduces the number of HBV-specific CD8^+^ T cells and secondary nonspecific infiltrates, preventing liver injury and fibrosis (Sitia et al., 2012). The pathophysiology of HBV infection and the role of platelet/T cell crosstalk are not fully understood. It has been proposed that interaction through platelet-CD40L and lymphocyte-CD40 (Elzey et al., 2003) could trigger an inflammatory endothelial response (Henn et al., 1998). Studies have shown an increased number of thrombotic events in patients with liver diseases (Tripodi and Mannucci, 2011), suggesting the potential for investigating antiplatelet drugs in further randomized clinical trials (RCTs) to assess their effects on HCC development in patients with chronic HBV infection.

### Platelets fuel cancer metastasis

Cancer metastasis, which involves the spread of cancer cells from a primary lesion to distant organs, is the main cause of cancer-related deaths (Massagué and Obenauf, 2016). Numerous studies have highlighted the crucial role platelets play in this process. Clinical observations have underlined the potential link between cancer cell diffusion via the bloodstream and platelet involvement in coagulation (Gay and Felding-Habermann, 2011; Contursi et al., 2017). Once cancer cells enter systemic circulation, they interact with platelets. Platelets can surround cancer cells, forming aggregates that promote survival, protect them from immune responses, and enhance their capacity to adhere to the endothelium, arrest, and exit blood vessels (Gay and Felding-Habermann, 2011).

Recent findings have shown that platelets induce a more malignant phenotype in cancer cells by enhancing their migratory properties (Labelle et al., 2011; Dovizio et al., 2013; Guillem-Llobat et al., 2016). Platelets release mediators such as PDGF, TGF-β, and PGE_2_, which promote EMT, that endows cancer cells with mesenchymal markers like vimentin and fibronectin and transcription factors such as Twist, Snail, and Zeb while downregulating epithelial markers like E-cadherin, thereby enabling these cells to acquire migratory properties and colonize distant organs (Kalluri and Weinberg, 2009). Labelle et al. (2011) demonstrated that EMT is induced in colon and breast cancer cells via platelet-derived TGF-β1, which activates the TGF-β/Smad pathway, thus promoting the metastatic potential of tumor cells. Dovizio et al. (2013) demonstrated that platelets trigger EMT and COX-2 overexpression in human adenocarcinoma cells (such as HT29 cells) through direct contact and PDGF release. The direct interaction involves the platelet collagen receptor GPVI and cancer cell galectin-3, unique among galectins due to its collagen-like domain. Inhibition of galectin-3 function [using β-lactose, a dominant-negative form of galectin-3, Gal-3C (Yang et al., 2008), or anti-galectin-3 antibody M3/38] or the inhibition of platelet adhesion to collagen-like binding sites on cancer cells by revacept, the soluble dimeric GPVI receptor fusion protein (GPVI-Fc) (Ungerer et al., 2011), prevented abnormal COX-2 expression. Revacept, which prevents the interaction between platelets and cancer cells, and the inhibition of COX-2 by rofecoxib, a highly selective COX-2 inhibitor, avoided platelet-induced changes in mRNA levels of EMT markers. This suggests that the direct platelet-cancer cell interaction and abnormal COX-2 expression contribute to the modifications in gene expression associated with EMT. Mammadova-Bach et al. (2020) found that genetic deficiency of platelet GPVI in mice reduced both experimental and spontaneous colon and breast cancer cell metastasis. Similar results were observed in mice lacking the spleen-tyrosine kinase Syk in platelets, a crucial component of the ITAM-signaling cascade. Both *in vitro* and *in vivo* analyses confirmed that mouse and human GPVI facilitated platelet adhesion to colon and breast cancer cells. Through a CRISPR/Cas9-based gene knockout approach, galectin-3 was identified as the primary counterreceptor of GPVI on tumor cells. Further, *in vivo* studies revealed that the interaction between platelet GPVI and tumor cell–expressed galectin-3 involves ITAM-signaling components in platelets and promotes the extravasation of tumor cells. Additionally, it was demonstrated that inhibiting GPVI by JAQ1 F (abʹ) 2 antibody efficiently impairs platelet–tumor cell interaction and tumor metastasis (Mammadova-Bach et al., 2020).

Guillem-Llobat et al. (2016) studied the impact of platelets on the spread of colorectal cancer cells (HT29 cells) to the lungs and the effect of the antiplatelet agent low-dose aspirin. When HT-29 cells were exposed to platelets *in vitro*, they were more likely to cause lung metastasis once injected in humanized immunodeficient mice than untreated HT29 cells. This effect was linked to the development of mesenchymal-like cancer cells by platelets associated with acquiring migratory properties and EMT. Mesenchymal-like cancer cells had an enhanced capacity to activate platelets, thus promoting the formation of platelet aggregates surrounding tumor cells, and this event is central in the development of cancer metastases. Administering a low dose of aspirin to the mice, prevented the increased rate of lung metastasis associated with the prevention of platelet activation *in vivo* in response to the injection of mesenchymal-like cancer cells. These results provide a mechanistic understanding of the reported antimetastatic properties of low-dose aspirin in posthoc analyses of randomized trials for cardiovascular prevention (Patrignani and Patrono, 2016) and reinforce the rationale for performing adjuvant trials of low-dose aspirin and possibly other antiplatelet agents, in colorectal cancer patients. *In vitro* studies of cocultures of platelets and HT29 cells showed that platelet-derived PGE_2_ activated the EP4 receptor on cancer cells, promoting EMT (Guillem-Llobat et al., 2016). Different antiplatelet agents, such as aspirin DG-041 (an EP3 receptor antagonist), and ticagrelor (a P2Y_12_ receptor antagonist), prevented EMT of cancer cells by inhibiting platelet activation and platelet-derived PGE_2_ release involved in cancer cell EP4-dependent migration (Guillem-Llobat et al., 2016).

Further studies investigated the potential of anticoagulant agents to disrupt communication between platelets and cancer cells. Battinelli et al. (2014) discovered that the interaction between breast cancer cells (MCF-7) and platelets stimulated the secretion of VEGF from platelets, a process that was prevented by the administration of low molecular weight heparin (LMWH) or fondaparinux. This suggests a thrombin-dependent release of angiogenic molecules by platelets after interaction with tumor cells. Human adenocarcinoma cell lines (such as HCT-8 and LoVo) and anaplastic murine tumor cells (such as Hut-20) can generate thrombin, which in turn activates platelets (Nierodzik and Karpatkin, 2006). Mitrugno et al. (2014) found that the colorectal cancer cell line Caco-2 and the prostate carcinoma cell line PC3M-luc induce platelet activation and granule secretion. They showed that the platelet immune receptor FcγRIIa plays a crucial role in this process by activating the FcγRIIa-spleen tyrosine kinase (Syk)-PLCγ signaling pathway, resulting in further platelet activation and granule secretion. This enhanced platelet recruitment and protection of circulating tumor cells from the immune system promote tumor cell survival and metastasis (Mitrugno et al., 2014), suggesting FcγRIIa as a target for antimetastatic therapy. Mannori et al. (1995) highlighted interactions between platelets and colon cancer cells (LS 180, T84, COLO 205, COLO 320, and HT-29) involving P-selectin on platelets and mucin-type glycoproteins on the cancer cell surface. Other studies reported the role of the platelet GPIIb/IIIa receptor in platelet/cancer cell crosstalk. In the melanoma cell line M3Dau, the interaction between the platelet receptor GPIIb/IIIa and a similar complex on tumor cells led to larger platelet-tumor aggregates (Boukerche et al., 1989). Mammadova-Bach et al. (2016) reported interactions between platelets and breast or colon tumor cells via platelet integrin α6β1 and ADAM-9 on tumor cells. This interaction promotes platelet activation, granule secretion, and endothelial transmigration, thus promoting metastasis. Genetic deletion of platelet α6β1 or an integrin α6-blocking antibody (GoH3) reduced lung metastasis by preventing platelet-tumor cell crosstalk (Mammadova-Bach et al., 2016). Lysophosphatidic acid (LPA), a platelet-derived mediator, plays a significant role in cancer development (Mills and Moolenaar, 2003). LPA acts through GPCRs, promoting platelet shape change and aggregation (Williams et al., 2009). Platelet-derived LPA promotes bone metastasis in ovarian and breast cancers by binding to LPA1 on tumor cells, and a specific LPA1 antagonist can inhibit this interaction without affecting normal platelet function (Boucharaba et al., 2004; 2006). Platelets also contribute to LPA biosynthesis via the enzyme autotaxin (ATX), which hydrolyzes LPA precursors to form LPA, binding to its receptors on cancer cells to promote invasion and metastasis (Leblanc et al., 2014). LPA can induce EMT in ovarian cancer cells by promoting nuclear translocation of β-catenin, activating Wnt/β-catenin target genes and hypoxia-induced factor-1α (HIF1α), leading to mesenchymal marker expression (Burkhalter et al., 2015; Ha et al., 2016).

The metabolite 12S-HETE, generated by platelet 12S-LOX, plays a crucial role in cancer development and metastasis through various mechanisms (Contursi et al., 2022). The involvement of 12S-LOX in cancer development has been demonstrated in human gastric cancer cells with *ALOX12* overexpression, and it is suggested as a marker of cancer progression in melanoma (Timár et al., 1999). 12-HETE is generated as a free acyl and is found in esterified form in membrane phospholipids, particularly in platelets where it is involved in coagulation (Thomas et al., 2010; Slatter et al., 2018; Lauder et al., 2017). HT29 cells do not express 12-LOX or synthesize 12-HETE when cultured alone, but in coculture with platelets, they express the protein and synthesize 12-HETE (Contursi et al., 2021). The interaction with cancer cells activates platelets that release EVs containing catalytically active 12S-LOX, quickly transferred to cancer cells. The cancer cells then acquire the capacity to generate 12-HETE, which is esterified rapidly, mainly in plasmalogen phospholipids. In cancer cells exposed to platelets, endogenous but not exogenous 12S-HETE contributed to changes in EMT gene expression by modifying cancer cell phospholipids by 12-HETE (Contursi et al., 2021). Modifying cancer cell phospholipids by 12S-HETE may functionally impact cancer cell biology, and the pharmacological inhibition of 12-LOX represents a novel anticancer strategy.

### Platelet-derived extracellular vesicles in cancer

Platelets secrete medium-size EVs (mEVs) (Gasecka et al., 2019; Kailashiya, 2018) ranging from 100 to 1,000 nm, released under various physiological and pathological conditions (Kailashiya, 2018). They are characterized by the inversion of membrane phospholipids, exposure of phosphatidylserine (PS) on the outer membrane—rendering them 50 to 100 times more procoagulant than platelets—and the expression of various integrins and enzymes (Janowska-Wieczorek et al., 2005; Italiano et al., 2010). These mEVs promote the communication between platelets and various cell types (Italiano et al., 2010). They act by (1) stimulating target cells through their surface ligands, (2) transferring surface receptors, and (3) delivering their cargo, i.e., proteins, bioactive lipids, mRNAs, transcription factors, and miRs, which can significantly alter cellular phenotypes. mEVs can also transfer infectious particles (e.g., HIV, prions) and intact organelles (e.g., mitochondria) (Ratajczak et al., 2006). Due to their carrier role, mEVs are involved in various biological processes, including hemostasis, thrombosis, inflammation, tumorigenesis, angiogenesis, and immunity (Italiano et al., 2010). For instance, platelet-derived EVs transfer receptors to both normal and cancer cells, enhancing adhesion, proliferation, and survival (e.g., CD41, CD61, CD62, CXCR4, PAR-1) (Baj-Krzyworzeka et al., 2002). mEVs also carry bioactive lipids like sphingosine-1-phosphate (S1P) and AA. Barry and colleagues demonstrated the transfer of platelet AA via mEVs to monocytes and endothelial cells, inducing COX-2 expression and prostanoid synthesis (Barry et al., 1997; 1999). Platelet mEVs transfer mRNA to monocytic THP-1 cells, increasing gamma-globin transcripts and hemoglobin subunits (Risitano et al., 2012). Additionally, platelet-derived mEVs contribute to tumorigenesis through miR transfer. For instance, the transfer of miR-223 to endothelial cells downregulates tumor-suppressor genes (Laffont et al., 2013), while the transfer of miR-939 to ovarian cancer cells promotes EMT (Tang et al., 2017). Conversely, mEVs carrying miR-24 induce apoptosis and suppress tumor growth (Michael et al., 2017). Platelet mEVs interact with monocytes/macrophages in inflammatory contexts, influencing proinflammatory cytokine release (Linke et al., 2017; Vasina et al., 2011).

Platelets contain important RNA biomarkers [mRNAs, miRs, circular (circ) RNAs, long non-coding (lnc) RNAs, mitochondrial-RNAs] and have a protein translation machinery for processing the RNA transcripts (Harrison and Goodall, 2008). CircRNAs, generated from exons of protein-coding genes through back-splicing, are abundant in platelets. They have received increasing attention for their potential role as cancer biomarkers due to their high stability and spatiotemporal-specific expression (Bach et al., 2019). In addition, lncRNAs (Dahariya et al., 2019) and miRs (Nagalla et al., 2011) represent important cancer biomarkers. Boerrigter et al. (2020) reported that the measurement of these molecules in prostate cancer (PCa) offers promising prospects because they are highly tumor-specific and relatively easy to detect. Through their crosstalk with other cells, including cancer cells, platelets can uptake these RNAs. These molecules can be found in isolated platelets. Analyzing RNAs from patients’ platelets offers significant promise for accurately distinguishing between metastatic and non-metastatic tumors. Additionally, RNA sequencing from platelets could identify the primary tumor location of six distinct tumor types with an accuracy of 71% (Hu et al., 2024).

Liquid biopsy involves analyzing biomarkers found in non-solid biological tissues, primarily blood. It offers significant advantages over traditional methods: it is risk-free, non-invasive, painless, does not necessitate surgery, and lowers costs and diagnostic time. The most extensively studied non-invasive cancer biomarkers include circulating tumor cells (CTCs), circulating tumor DNA (ctDNA), and EV cargo (Marrugo-Ramírez et al., 2018). They offer several advantages, including the ability for early detection, assessment of an individual patient’s prognosis, such as cancer stage and spread, identification of new targets for personalized treatments, and predict therapy responses. Currently, blood-based biopsy measurements focus on the evaluation of biomarker biosources, including circulating tumor DNA (ctDNA), circulating tumor cells (CTCs), EVs (i.e., exosomes, mEVs and oncosomes), and tumor-educated platelets (TEPs) (Marrugo-Ramírez et al., 2018; Best et al., 2015; Nilsson et al., 2011; 2016; Skog et al., 2008).

Contursi et al. (2023) studied platelet-derived mEVs collected from non-metastatic CRC patients at initial diagnosis versus healthy controls (HCs). The aim was to address how these mEVs can change the expression of genes related to the process of EMT in four different human colorectal cancer cell lines: HCA7, HCT116, HT29, and Caco2. These cell lines have different genetic features and metastatic potentials. The study found that platelet-derived mEVs from CRC patients affect colorectal cancer cells differently depending on their genetic makeup and behavior. Specifically, the mEVs from CRC patients caused an increase in the expression of *TWIST1* and *VIM* (protein name: vimentin) in all the studied cancer cell lines, while those from healthy subjects did not. In HCA7 cells, the expression of *CDH1* (protein name, E-cadherin) was not affected by the mEVs from either CRC patients or HCs. However, the HCT116, HT29, and Caco2 cells showed decreased *CDH1* expression when cultured with mEVs from CRC patients, with the HCT116 cells (which have high metastatic potential) showing decreased expression after just 4 hours of exposure. Moreover, the mEVs from CRC patients and HCs induced the expression of COX-2 in colorectal cancer cell lines at 24 but not at 4 hours, suggesting that this effect may be due to post-transcriptional regulation (Contursi et al., 2023).

Cancer-associated thrombosis is a leading cause of death in cancer patients (Mahajan A. et al., 2022). Contursi et al. (2023) explored whether platelet-derived mEVs collected from CRC patients affected the TXB_2_ biosynthesis of 4 CRC cell lines *in vitro*. Proteomic analysis revealed that COX-1 and TXA_2_ synthase were expressed similarly in mEVs collected from CRC patients and healthy controls (HC). HCA7 cells can generate endogenously TXB_2_, and when exposed to mEVs, the TXB_2_ levels were unchanged. Other CRC cell lines have very limited capacity to generate TXB_2_, but when cocultured with mEVs from both CRC patients and HCs, TXB_2_ was detectable. This may suggest that platelet-derived mEVs could provide AA to cancer cells, leading to TXB_2_ generation. Alternatively, platelet-derived mEVs might transfer COX-1 and/or TXA_2_ synthase to cancer cells, allowing them to produce TXB_2_.

A comparison of the proteomic profiles of mEVs from CRC patients and HCs revealed 208 significantly altered proteins. High expression of HLA-B class I and PSMD2 in CRC patient mEVs was detected. The human leukocyte antigen (HLA) system or complex is a group of related proteins encoded by the major histocompatibility complex (MHC) gene; it has been reported that cell surface expression of HLA I/MHC I molecules is a marker of young, reactive platelets (Angénieux et al., 2019). PSMD2 is part of the ubiquitin-proteasome system and plays a role in immune modulation (Colberg et al., 2020). Platelets contain proteasome and immunoproteasome components, allowing them to process foreign proteins into peptide fragments. These fragments are then loaded onto MHC I molecules and presented on the surface of the platelets as peptide-MHC I complexes. Immunoproteasomes may regulate platelets’ involvement in innate and adaptive immunity, highlighting the connection between hemostasis and inflammation. The increased protein levels of HLA-B class I and PSMD2 found in platelet-derived mEVs from CRC patients by Contursi et al. (2023) suggest that platelet-derived mEVs are modulators of the immune response in CRC. A comprehensive examination of the protein composition and its alterations associated with cancer underscores the potential of platelet-derived mEV proteomics to facilitate early diagnosis, provide continuous monitoring, and support personalized treatment strategies, ultimately enhancing patient outcomes.

## Conclusion

The complex and intricate interplay between inflammation and platelets in the development of tumors presents exciting opportunities for pioneering new avenues in cancer prevention and treatment strategies. A wealth of clinical and experimental evidence underscores platelets’ pivotal and multifaceted role in fostering an inflammatory microenvironment that fuels tumor growth and progression. Moreover, platelets play a key role in the development of cancer metastasis. This huge amount of knowledge opens the way to using antiplatelet agents, such as low-dose aspirin, which exhibit considerable potential in dampening platelet activation and consequent inflammatory processes, thereby lowering cancer risk, particularly in cases of CRC (Patrignani and Patrono, 2016; 2018). It is necessary to investigate further the effectiveness of alternative antiplatelet medications, such as ADP P2Y_12_ receptor antagonists (Ballerini et al., 2018). Other potential new antiplatelet drugs, such as revacept (Ungerer et al., 2011; Mayer et al., 2021) and selective 12-LOX inhibitors (Tourdot and Holinstat, 2017), currently under clinical development, are of interest due to their lower risk of bleeding (Sim et al., 2023).

The role of platelets in promoting the initial stages of cancer development is supported by various evidence, including studies using knockout mouse models and clinical research (Bruno et al., 2022; Patrignani and Patrono, 2016). Platelets contribute to inflammation by releasing various molecules and EVs. The molecules delivered by platelets activate different signaling pathways in the cells involved in inflammation and immunity (Patrignani and Patrono, 2018). Platelets are essential for cell-cell communication and significantly influence the phenotype of cellular components within the stromal compartment of tissues. An emerging and continuously evolving paradigm underscores the synergistic connection between cardiovascular disease (CVD) and cancer, elucidated by their shared modifiable risk factors, which encompass tobacco use, obesity, diabetes mellitus, dietary patterns, physical activity, and alcohol consumption. It is important to note that all these factors contribute to the activation of platelets, making this response a key event in both cancer and cardiovascular disease (Patrignani and Patrono, 2016; Davì and Patrono, 2007). This helps explain why using antiplatelet agents like low-dose aspirin effectively prevents cardiovascular disease and possibly cancer (Patrignani and Patrono, 2016). Platelet activation results in an increased generation of TXA_2_ and PGE_2_, subsequently triggering the activation of immune cells, fibroblasts, and endothelial cells. Thus, the inhibition of platelet function effectively impedes the release of molecules from platelets involved in initial events linked to tumorigenesis (Patrignani and Patrono, 2016). In cases where cancer has already developed, antiplatelet agents are likely less effective because they have limited capacity to directly affect the cellular component of a cancerous lesion (Patrignani et al., 2024). However, platelets can infiltrate cancerous tissues (Bruno et al., 2022), which can lead to tumor immune evasion, inflammation, and EMT (Patrignani and Patrono, 2016; 2018). This suggests that antiplatelet agents could indirectly cause antitumor effects by reducing platelet accumulation in tumors.

Genetic alterations may affect an individual’s response to aspirin. However, Frouws et al. (2017) reported that the mutation status of the BRAF and KRAS genes should not be regarded as reliable indicators for personalized aspirin therapy. In contrast, Liao et al. (2012) showed that tumors with mutations in the PIK3CA gene (which codes for the protein phosphatidylinositol 4,5-bisphosphate 3-kinase catalytic subunit alpha isoform) exhibit increased sensitivity to the effects of aspirin. Nonetheless, the underlying mechanisms explaining this association remain to be elucidated.

Ongoing clinical trials investigate aspirin’s potential as an adjuvant cancer therapy in various doses and durations in CRC patients (Table 1). Some of these trials are targeting patients based on specific biomarkers. These trials are part of the Prospective Aspirin Meta-analysis, registered on PROSPERO in October 2023. The main objective of this meta-analysis is to ascertain the potential moderate effects of aspirin, both overall and within different subgroups. These subgroups include those defined by tumor location (right-sided versus left-sided) and specific biomarkers.

**TABLE 1:** Ongoing Phase III placebo-controlled trials for aspirin in adjuvant colorectal cancer.

Trial (Clinical trials ID)	Patients (number)	Aspirin dose (duration)	Primary/Secondary endpoint	Actual status
Add-Aspirin (NCT02804815)	Stage II/III or IV CRC with completely resected liver metastasis (2332)	100 mg and 300 mg (5 years)	DFS/OS	Recruiting
ASCOLT (NCT00565708)	Dukes C and high-risk B colon and rectal cancers (1587)	200 mg (3 years)	DFS/OS	Active, not recruiting
EPISODE-III (Japan Registry of Clinical Trials as jRCTs031180009)	Stage III CRC following curative resection (880)	100 mg (3 years)	DFS/OS	Recruiting
ASPIRIN (NCT02301286, NCT03464305)	Stage II/III colon cancer in Netherlands and Belgium (770)	80 mg (5years)	DFS/OS	Active, not recruiting
ALASCCA (NCT02647099)	Stage II-III CRC with PI3K mutation (627)	160 mg (3 years)	DFS/OS	Active, not recruiting
ASAC (NCT03326791)	CRC with resected liver metastasis (446)	160 mg (3years)	DFS/OS	Active, not recruiting
ASPIK (NCT02945033)	Stage II-III CRC with PI3K mutation (134)	100 mg (3 years)	DFS/OS	Recruiting
SAKK 41/13 (NCT02467582)	Stage II-III CRC with PI3K mutation (113)	300 mg (3years)	DFS/OS	Active, not recruiting

Aspirin for Dukes’ C and high-risk Dukes’ B colorectal cancers (ASCOLT); Efficacy of aspirin for stage III, colorectal cancer: a randomized double-blind placebo-controlled trial (EPISODE III); Adjuvant Low Dose Aspirin in Colorectal Cancer (ALASCCA); Aspirin in Colorectal Cancer Liver Metastases (ASAC); Aspirin Versus Placebo in Resected Colon Cancer With PI3K Mutation Stage III, or II, High Risk (ASPIK); Colorectal cancer (CRC); Disease free survival (DFS); Overall survival (OS).

At long follow-up, aspirin (300 mg BID) has been shown to prevent Lynch Syndrome (LS) cancers, including CRC (Burn et al., 2008; 2011; 2020). LS is a dominantly inherited cancer predisposition syndrome characterized by an increased risk of numerous cancers. LS-associated tumors are mismatch repair deficient (Engel et al., 2020). Aspirin is now recommended in NICE guidelines to prevent CRC in LS (National Institute for Health and Care, 2020; Serrano et al., 2022). CaPP3 trial (https://www.capp3.org/) is ongoing to define the most appropriate aspirin dose in LS. The trial is a non-inferiority study comparing daily aspirin of 600, 300, and 100 mg (100 mg/d is the dose recommended for preventing CVD). The finding of comparable efficacy of the higher doses versus the low dose of aspirin (100 mg/d) would support the platelet hypothesis in tumorigenesis (Patrignani and Patrono, 2016; 2018), thus opening the way to its use for cancer prevention in LS.

However, chronic aspirin use, even at low doses, can be associated with increased bleeding (Patrono and Baigent, 2019), especially in older individuals. Therefore, the decision to treat individual patients with low-dose aspirin should consider the bleeding risk weighed against CVD prevention and cancer benefits.

In the current era of precision medicine, developing treatment protocols involving the safe utilization of antiplatelet agents to prevent cancer and cardiovascular disease necessitates adopting a systems biology approach. This strategy entails a comprehensive analysis of heterogeneous datasets, including genomics, epigenomics, proteomics, lipidomics, and clinical data, at the level of the individual patient. This procedure involves dynamic systems modeling to identify candidate pathways that contribute to the benefits and harms of aspirin and possibly other antiplatelet agents. Additionally, this strategy will help identify susceptibility profiles for CRC and possibly other types of cancer, verifying whether platelet- and EV-based liquid biopsy can predict the onset and recurrence of cancer.

## References

[B1] Adili B. E. T. Mast K. Yeung J. Freedman J. C. Green A. Luci D. K. (2017). First selective 12-LOX inhibitor, ML355, impairs thrombus formation and vessel occlusion *in vivo* with minimal effects on hemostasis. Arterioscler. Thromb. Vasc. Biol. 37, 1828–1839. 10.1161/ATVBAHA.117.309868 28775075 PMC5620123

[B2] Ahmed I. Majeed A. Powell R. (2007). Heparin induced thrombocytopenia: diagnosis and management update. Postgrad. Med. J. 83 (983), 575–582. 10.1136/pgmj.2007.059188 17823223 PMC2600013

[B3] Ali R. A. Wuescher L. M. Worth R. G. (2015). Platelets: essential components of the immune system. Curr. Trends Immunol. 16, 65–78. 27818580 PMC5096834

[B4] Angénieux C. Dupuis A. Gachet C. de la Salle H. Maître B. (2019). Cell surface expression of HLA I molecules as a marker of young platelets. J. Thromb. Haemost. 17 (9), 1511–1521. 10.1111/jth.14537 31207003

[B5] Arepally G. M. Padmanabhan A. (2021). Heparin-induced thrombocytopenia: a focus on thrombosis. Arterioscler. Thromb. Vasc. Biol. 41 (1), 141–152. 10.1161/ATVBAHA.120.315445 33267665 PMC7769912

[B6] Arman M. Krauel K. (2015). Human platelet IgG Fc receptor FcγRIIA in immunity and thrombosis. J. Thromb. Haemost. 13 (6), 893–908. 10.1111/jth.12905 25900780

[B7] Bach D. H. Lee S. K. Sood A. K. (2019). Circular RNAs in cancer. Mol. Ther. Nucleic Acids 16, 118–129. 10.1016/j.omtn.2019.02.005 30861414 PMC6411617

[B8] Baj-Krzyworzeka M. Majka M. Pratico D. Ratajczak J. Vilaire G. Kijowski J. (2002). Platelet-derived microparticles stimulate proliferation, survival, adhesion, and chemotaxis of hematopoietic cells. Exp. Hematol. 30, 450–459. 10.1016/s0301-472x(02)00791-9 12031651

[B9] Balkwill F. Charles K. A. Mantovani A. (2005). Smoldering and polarized inflammation in the initiation and promotion of malignant disease. Cancer Cell 7 (3), 211–217. 10.1016/j.ccr.2005.02.013 15766659

[B10] Ballerini P. Dovizio M. Bruno A. Tacconelli S. Patrignani P. (2018). P2Y_12_ receptors in tumorigenesis and metastasis. Front. Pharmacol. 9, 66. 10.3389/fphar.2018.00066 29456511 PMC5801576

[B11] Barron D. A. Rowley D. R. (2012). The reactive stroma microenvironment and prostate cancer progression. Endocr. Relat. Cancer 19 (6), R187–R204. 10.1530/ERC-12-0085 22930558 PMC3716392

[B12] Barry O. P. Kazanietz M. G. Praticò D. FitzGerald G. A. (1999). Arachidonic acid in platelet microparticles up-regulates cyclooxygenase-2-dependent prostaglandin formation via a protein kinase C/mitogen-activated protein kinase-dependent pathway. J. Biol. Chem. 274, 7545–7556. 10.1074/jbc.274.11.7545 10066822

[B13] Barry O. P. Pratico D. Lawson J. A. FitzGerald G. A. (1997). Transcellular activation of platelets and endothelial cells by bioactive lipids in platelet microparticles. J. Clin. Investigation 99, 2118–2127. 10.1172/JCI119385 PMC508042 9151784

[B14] Battinelli E. M. Markens B. A. Kulenthirarajan R. A. Machlus K. R. Flaumenhaft R. Italiano J. E. Jr (2014). Anticoagulation inhibits tumor cell-mediated release of platelet angiogenic proteins and diminishes platelet angiogenic response. Blood 123 (1), 101–112. 10.1182/blood-2013-02-485011 24065244 PMC3879901

[B15] Bertagnolli M. M. Eagle C. J. Zauber A. G. Redston M. Solomon S. D. Kim K. (2006). Celecoxib for the prevention of sporadic colorectal adenomas. N. Engl. J. Med. 355 (9), 873–884. 10.1056/NEJMoa061355 16943400

[B16] Best M. G. Sol N. Kooi I. Tannous J. Westerman B. A. Rustenburg F. (2015). RNA-seq of tumor-educated platelets enables blood-based pan-cancer, multiclass, and molecular pathway cancer diagnostics. Cancer Cell 28 (5), 666–676. 10.1016/j.ccell.2015.09.018 26525104 PMC4644263

[B17] Boerrigter E. Groen L. N. Van Erp N. P. Verhaegh G. W. Schalken J. A. (2020). Clinical utility of emerging biomarkers in prostate cancer liquid biopsies. Expert Rev. Mol. Diagn 20 (2), 219–230. 10.1080/14737159.2019.1675515 31577907

[B18] Boucharaba A. Serre C. M. Grès S. Saulnier-Blache J. S. Bordet J. C. Guglielmi J. (2004). Platelet-derived lysophosphatidic acid supports the progression of osteolytic bone metastases in breast cancer. J. Clin. Invest 114, 1714–1725. 10.1172/JCI22123 15599396 PMC535068

[B19] Boucharaba A. Serre C. M. Guglielmi J. Bordet J. C. Clézardin P. Peyruchaud O. (2006). The type 1 lysophosphatidic acid receptor is a target for therapy in bone metastases. Proc. Natl. Acad. Sci. U. S. A. 103, 9643–9648. 10.1073/pnas.0600979103 16769891 PMC1480460

[B20] Boukerche H. Berthier-Vergnes O. Tabone E. Doré J. F. Leung L. L. McGregor J. L. (1989). Platelet-melanoma cell interaction is mediated by the glycoprotein IIb-IIIa complex. Blood 74, 658–663. 10.1182/blood.v74.2.658.bloodjournal742658 2752140

[B21] Boulaftali Y. Hess P. R. Kahn M. L. Bergmeier W. (2014). Platelet immunoreceptor tyrosine-based activation motif (ITAM) signaling and vascular integrity. Circ. Res. 114 (7), 1174–1184. 10.1161/CIRCRESAHA.114.301611 24677237 PMC4000726

[B22] Boylan B. Gao C. Rathore V. Gill J. C. Newman D. K. Newman P. J. (2008). Identification of FcgammaRIIa as the ITAM-bearing receptor mediating alphaIIbbeta3 outside-in integrin signaling in human platelets. Blood 112 (7), 2780–2786. 10.1182/blood-2008-02-142125 18641368 PMC2556613

[B23] Bruno A. Contursi A. Tacconelli S. Sacco A. Hofling U. Mucci M. (2022). The specific deletion of cyclooxygenase-1 in megakaryocytes/platelets reduces intestinal polyposis in Apc^Min/+^ mice. Pharmacol. Res. 185, 106506. 10.1016/j.phrs.2022.106506 36241001

[B24] Burkhalter R. J. Westfall S. D. Liu Y. Stack M. S. (2015). Lysophosphatidic acid initiates epithelial to mesenchymal transition and induces β-Catenin-mediated transcription in epithelial ovarian carcinoma. J. Biol. Chem. 290, 22143–22154. 10.1074/jbc.M115.641092 26175151 PMC4571965

[B25] Burn J. Bishop D. T. Mecklin J. P. Macrae F. Möslein G. Olschwang S. (2008). Effect of aspirin or resistant starch on colorectal neoplasia in the Lynch syndrome. N. Engl. J. Med. 359 (24), 2567–2578. 10.1056/NEJMoa0801297 19073976

[B26] Burn J. Gerdes A. M. Macrae F. Mecklin J. P. Moeslein G. Olschwang S. (2011). Long-term effect of aspirin on cancer risk in carriers of hereditary colorectal cancer: an analysis from the CAPP2 randomised controlled trial. Lancet 378 (9809), 2081–2087. 10.1016/S0140-6736(11)61049-0 22036019 PMC3243929

[B27] Burn J. Sheth H. Elliott F. Reed L. Macrae F. Mecklin J. P. (2020). Cancer prevention with aspirin in hereditary colorectal cancer (Lynch syndrome), 10-year follow-up and registry-based 20-year data in the CAPP2 study: a double-blind, randomised, placebo-controlled trial. Lancet 395 (10240), 1855–1863. 10.1016/S0140-6736(20)30366-4 32534647 PMC7294238

[B28] Celis P. M. Celis A. Nielsen H. V. Gesser B. (1987). Cyclin (PCNA, auxiliary protein of DNA polymerase delta) is a central component of the pathway(s) leading to DNA replication and cell division. FEBS Lett. 220, 1–7. 10.1016/0014-5793(87)80865-7 2886367

[B29] Chen L. Deng H. Cui H. Fang J. Zuo Z. Deng J. (2017). Inflammatory responses and inflammation-associated diseases in organs. Oncotarget 9 (6), 7204–7218. 10.18632/oncotarget.23208 29467962 PMC5805548

[B30] Chitu V. Stanley E. R. (2006). Colony-stimulating factor-1 in immunity and inflammation. Curr. Opin. Immunol. 18 (1), 39–48. 10.1016/j.coi.2005.11.006 16337366

[B31] Cloutier N. Allaeys I. Marcoux G. Machlus K. R. Mailhot B. Zufferey A. (2018). Platelets release pathogenic serotonin and return to circulation after immune complex-mediated sequestration. PNAS 115, E1550-E1559–E1559. 10.1073/pnas.1720553115 29386381 PMC5816207

[B32] Coenen D. M. Heinzmann A. C. A. Karel M. F. A. Cosemans JMEM Koenen R. R. (2021). The multifaceted contribution of platelets in the emergence and aftermath of acute cardiovascular events. Atherosclerosis 319, 132–141. 10.1016/j.atherosclerosis.2020.12.017 33468314

[B33] Colberg L. Cammann C. Greinacher A. Seifert U. (2020). Structure and function of the ubiquitin-proteasome system in platelets. J. Thromb. Haemost. 18, 771–780. 10.1111/jth.14730 31898400

[B34] Contursi A. Fullone R. Szklanna-Koszalinska P. Marcone S. Lanuti P. Taus F. (2023). Tumor-educated platelet extracellular vesicles: proteomic profiling and crosstalk with colorectal cancer cells. Cancers (Basel) 15 (2), 350. 10.3390/cancers15020350 36672299 PMC9856452

[B35] Contursi A. Sacco A. Grande R. Dovizio M. Patrignani P. (2017). Platelets as crucial partners for tumor metastasis: from mechanistic aspects to pharmacological targeting. Cell. Mol. Life Sci. 74, 3491–3507. 10.1007/s00018-017-2536-7 28488110 PMC11107532

[B36] Contursi A. Schiavone S. Dovizio M. Hinz C. Fullone R. Tacconelli S. (2021). Platelets induce free and phospholipid-esterified 12-hydroxyeicosatetraenoic acid generation in colon cancer cells by delivering 12-lipoxygenase. J. Lipid Res. 62, 100109. 10.1016/j.jlr.2021.100109 34428433 PMC8456051

[B37] Contursi A. Tacconelli S. Hofling U. Bruno A. Dovizio M. Ballerini P. (2022). Biology and pharmacology of platelet-type 12-lipoxygenase in platelets, cancer cells, and their crosstalk. Biochem. Pharmacol. 205, 115252. 10.1016/j.bcp.2022.115252 36130648

[B38] Coussens L. M. Werb Z. (2002). Inflammation and cancer. Nature 420 (6917), 860–867. 10.1038/nature01322 12490959 PMC2803035

[B39] D’Agostino I. Tacconelli S. Bruno A. Contursi A. Mucci L. Hu X. (2021). Low-dose Aspirin prevents hypertension and cardiac fibrosis when thromboxane A_2_ is unrestrained. Pharmacol. Res. 170, 105744. 10.1016/j.phrs.2021.105744 34182131

[B40] Dahariya S. Paddibhatla I. Kumar S. Raghuwanshi S. Pallepati A. Gutti R. K. (2019). Long non-coding RNA: classification, biogenesis and functions in blood cells. Mol. Immunol. 112, 82–92. 10.1016/j.molimm.2019.04.011 31079005

[B41] Danese S. Motte Cd C. Fiocchi C. (2004). Platelets in inflammatory bowel disease:clinical, pathogenic, and therapeutic implications. Am. J. Gastroenterol. 99, 938–945. 10.1111/j.1572-0241.2004.04129.x 15128364

[B42] Dang T. O. Ogunniyi A. Barbee M. S. Drilon A. (2016). Pembrolizumab for the treatment of PD-L1 positive advanced or metastatic non-small cell lung cancer. Expert Rev. Anticancer Ther. 16 (1), 13–20. 10.1586/14737140.2016.1123626 26588948 PMC4993158

[B43] Davì G. Patrono C. (2007). Platelet activation and atherothrombosis. N. Engl. J. Med. 357 (24), 2482–2494. 10.1056/NEJMra071014 18077812

[B44] De La Cruz A. Hargrave A. Magadi S. Courson J. A. Landry P. T. Zhang W. (2021). Platelet and erythrocyte extravasation across inflamed corneal venules depend on CD18, neutrophils, and mast cell degranulation. Int. J. Mol. Sci. 22 (14), 7360. 10.3390/ijms22147360 34298979 PMC8329926

[B45] Delves P. J. Roitt I. M. (2000). The immune system First of two parts. N. Engl. J. Med. 343 (1), 37–49. 10.1056/NEJM200007063430107 10882768

[B46] Diacovo T. G. deFougerolles A. R. Bainton D. F. Springer T. A. (1994). A functional integrin ligand on the surface of platelets: intercellular adhesion molecule-2. J. Clin. Invest 94 (3), 1243–1251. 10.1172/JCI117442 8083366 PMC295209

[B47] Dovizio M. Alberti S. Guillem-Llobat P. Patrignani P. (2014). Role of platelets in inflammation and cancer: novel therapeutic strategies. Basic Clin. Pharmacol. Toxicol. 114 (1), 118–127. 10.1111/bcpt.12156 24118902

[B48] Dovizio M. Maier T. J. Alberti S. Di Francesco L. Marcantoni E. Munch G. (2013). Pharmacological inhibition of platelet-tumor cell cross-talk prevents platelet-induced overexpression of cyclooxygenase-2 in HT29 human colon carcinoma cells. Mol. Pharmacol. 84, 25–40. 10.1124/mol.113.084988 23580446 PMC11037430

[B49] Dunn G. P. Bruce A. T. Ikeda H. Old L. J. Schreiber R. D. (2002). Cancer immunoediting: from immunosurveillance to tumor escape. Nat. Immunol. 3 (11), 991–998. 10.1038/ni1102-991 12407406

[B50] Elzey B. D. Tian J. Jensen R. J. Swanson A. K. Lees J. R. Lentz S. R. (2003). Platelet-mediated modulation of adaptive immunity. A communication link between innate and adaptive immune compartments. Immunity 19, 9–19. 10.1016/s1074-7613(03)00177-8 12871635

[B51] Engel C. Ahadova A. Seppälä T. T. Aretz S. Bigirwamungu-Bargeman M. Bläker H. German HNPCC Consortium, the Dutch Lynch Syndrome Collaborative Group (2020). Associations of pathogenic variants in MLH1, MSH2, and MSH6 with risk of colorectal adenomas and tumors and with somatic mutations in patients with Lynch syndrome. Gastroenterology 158 (5), 1326–1333. 10.1053/j.gastro.2019.12.032 31926173

[B52] Evangelista V. Manarini S. Sideri R. Rotondo S. Martelli N. Piccoli A. (1999). Platelet/polymorphonuclear leukocyte interaction: P-selectin triggers protein-tyrosine phosphorylation-dependent CD11b/CD18 adhesion: role of PSGL-1 as a signaling molecule. Blood 93 (3), 876–885. 10.1182/blood.v93.3.876.403k25_876_885 9920836

[B53] Faour W. H. He Y. He Q. W. de Ladurantaye M. Quintero M. Mancini A. (2001). Prostaglandin E(2) regulates the level and stability of cyclooxygenase-2 mRNA through activation of p38 mitogen-activated protein kinase in interleukin-1 beta-treated human synovial fibroblasts. J. Biol. Chem. 276 (34), 31720–31731. 10.1074/jbc.M104036200 11423555

[B54] Frouws M. A. Reimers M. S. Swets M. Bastiaannet E. Prinse B. van Eijk R. (2017). The influence of BRAF and KRAS mutation status on the association between aspirin use and survival after colon cancer diagnosis. PLoS One 12 (1), e0170775. 10.1371/journal.pone.0170775 28125730 PMC5268402

[B55] Gasecka A. Nieuwland R. van der Pol E. Hajji N. Ćwiek A. Pluta K. (2019). P2Y12 antagonist ticagrelor inhibits the release of procoagulant extracellular vesicles from activated platelets. Cardiol. J. 26 (6), 782–789. 10.5603/CJ.a2018.0045 29671861 PMC8083044

[B56] Gawaz M. Geisler T. Borst O. (2023). Current concepts and novel targets for antiplatelet therapy. Nat. Rev. Cardiol. 20 (9), 583–599. 10.1038/s41569-023-00854-6 37016032

[B57] Gawaz M. Langer H. May A. E. (2005). Platelets in inflammation and atherogenesis. J. Clin. Invest 115, 3378–3384. 10.1172/JCI27196 16322783 PMC1297269

[B58] Gay L. J. Felding-Habermann B. (2011). Contribution of platelets to tumour metastasis. Nat. Rev. Cancer 11, 123–134. 10.1038/nrc3004 21258396 PMC6894505

[B59] Golebiewska Poole A. W. (2015). Platelet secretion: from haemostasis to wound healing and beyond. Blood Rev. 29, 153–162. 10.1016/j.blre.2014.10.003 25468720 PMC4452143

[B60] Goodall A. H. (2014). Platelet 12-LOX scores a HIT. Blood 124 (14), 2166–2168. 10.1182/blood-2014-08-595652 25278566

[B61] Gresele P. Falcinelli E. Momi S. Petito E. Sebastiano M. (2021). Platelets and matrix metalloproteinases: a bidirectional interaction with multiple pathophysiologic implications. Hamostaseologie 41 (2), 136–145. 10.1055/a-1393-8339 33860521

[B62] Grosser T. Fries S. FitzGerald G. A. (2006). Biological basis for the cardiovascular consequences of COX-2 inhibition: therapeutic challenges and opportunities. J. Clin. Invest 116 (1), 4–15. 10.1172/JCI27291 16395396 PMC1323269

[B63] Guidotti L. G. Chisari F. V. (2006). Immunobiology and pathogenesis of viral hepatitis. Annu. Rev. Pathology 1, 23–61. 10.1146/annurev.pathol.1.110304.100230 18039107

[B64] Guillem-Llobat P. Dovizio M. Bruno A. Ricciotti E. Cufino V. Sacco A. (2016). Aspirin prevents colorectal cancer metastasis in mice by splitting the crosstalk between platelets and tumor cells. Oncotarget 7, 32462–32477. 10.18632/oncotarget.8655 27074574 PMC5078026

[B65] Ha J. H. Ward J. D. Radhakrishnan R. Jayaraman M. Song Y. S. Dhanasekaran D. N. (2016). Lysophosphatidic acid stimulates epithelial to mesenchymal transition marker Slug/Snail2 in ovarian cancer cells via Gαi2, Src, and HIF1α signaling nexus. Oncotarget 7, 37664–37679. 10.18632/oncotarget.9224 27166196 PMC5122340

[B66] Harrison P. Goodall A. H. (2008). Message in the platelet-more than just vestigial mRNA. Platelets 19 (6), 395–404. 10.1080/09537100801990582 18925506

[B67] Henn V. Slupsky J. R. Gräfe M. Anagnostopoulos I. Förster R. Müller-Berghaus G. (1998). CD40 ligand on activated platelets triggers an inflammatory reaction of endothelial cells. Nature 391, 591–594. 10.1038/35393 9468137

[B68] Hinterleitner C. Strähle J. Malenke E. Hinterleitner M. Henning M. Seehawer M. (2021). Platelet PD-L1 reflects collective intratumoral PD-L1 expression and predicts immunotherapy response in non-small cell lung cancer. Nat. Commun. 12 (1), 7005. 10.1038/s41467-021-27303-7 34853305 PMC8636618

[B69] Hu H. Song H. Han B. Zhao H. He J. (2024). Tumor-educated platelet RNA and circulating free RNA: emerging liquid biopsy markers for different tumor types. Front. Biosci. Landmark Ed. 29 (2), 80. 10.31083/j.fbl2902080 38420812

[B70] Hussain S. P. Harris C. C. (2007). Inflammation and cancer: an ancient link with novel potentials. Int. J. Cancer 121 (11), 2373–2380. 10.1002/ijc.23173 17893866

[B71] Italiano J. E. Jr Mairuhu A. T. Flaumenhaft R. (2010). Clinical relevance of microparticles from platelets and megakaryocytes. Curr. Opin. Hematol. 17, 578–584. 10.1097/MOH.0b013e32833e77ee 20739880 PMC3082287

[B72] Janowska-Wieczorek A. Wysoczynski M. Kijowski J. Marquez-Curtis L. Machalinski B. Ratajczak J. (2005). Microvesicles derived from activated platelets induce metastasis and angiogenesis in lung cancer. Int. J. Cancer 113, 752–760. 10.1002/ijc.20657 15499615

[B73] Joung J. K. Sander J. D. (2013). TALENs: a widely applicable technology for targeted genome editing. Nat. Rev. Mol. Cell Biol. 14, 49–55. 10.1038/nrm3486 23169466 PMC3547402

[B74] Juneja V. R. McGuire K. A. Manguso R. T. LaFleur M. W. Collins N. Haining W. N. (2017). PD-L1 on tumor cells is sufficient for immune evasion in immunogenic tumors and inhibits CD8 T cell cytotoxicity. J. Exp. Med. 214, 895–904. 10.1084/jem.20160801 28302645 PMC5379970

[B75] Kailashiya J. (2018). Platelet-derived microparticles analysis: techniques, challenges and recommendations. Anal. Biochem. 546, 78–85. 10.1016/j.ab.2018.01.030 29408673

[B76] Kalluri R. Weinberg R. A. (2009). The basics of epithelial-mesenchymal transition. J. Clin. Investigation 119, 1420–1428. 10.1172/JCI39104 PMC2689101 19487818

[B77] Kalluri R. Zeisberg M. (2006). Fibroblasts in cancer. Nat. Rev. Cancer 6 (5), 392–401. 10.1038/nrc1877 16572188

[B78] Kitamura T. Qian B. Z. Pollard J. W. (2015). Immune cell promotion of metastasis. Nat. Rev. Immunol. 15, 73–86. 10.1038/nri3789 25614318 PMC4470277

[B79] Labelle M. Begum S. Hynes R. O. (2011). Direct signaling between platelets and cancer cells induces an epithelial-mesenchymal-like transition and promotes metastasis. Cancer Cell 20, 576–590. 10.1016/j.ccr.2011.09.009 22094253 PMC3487108

[B80] Laffont B. Corduan A. Plé H. Duchez A. C. Cloutier N. Boilard E. (2013). Activated platelets can deliver mRNA regulatory Ago2⋅microRNA complexes to endothelial cells via microparticles. Blood 122, 253–261. 10.1182/blood-2013-03-492801 23652806

[B81] Lauder S. N. Allen-Redpath K. Slatter D. A. Aldrovandi M. O'Connor A. Farewell D. (2017). Networks of enzymatically oxidized membranelipids support calcium-dependent coagulation factor binding to maintain hemostasis. Sci. Signal. 10, eaan2787. 10.1126/scisignal.aan2787 29184033 PMC5720345

[B82] Leblanc R. Lee S. C. David M. Bordet J. C. Norman D. D. Patil R. (2014). Interaction of platelet-derived autotaxin with tumor integrin αVβ3 controls metastasis of breast cancer cells to bone. Blood 124, 3141–3150. 10.1182/blood-2014-04-568683 25277122 PMC4231421

[B83] Li S. Jiang M. Wang L. Yu S. (2020). Combined chemotherapy with cyclooxygenase-2 (COX-2) inhibitors in treating human cancers: recent advancement. Biomed. Pharmacother. 129, 110389. 10.1016/j.biopha.2020.110389 32540642

[B84] Liao X. Lochhead P. Nishihara R. Morikawa T. Kuchiba A. Yamauchi M. (2012). Aspirin use, tumor PIK3CA mutation, and colorectal-cancer survival. N. Engl. J. Med. 367 (17), 1596–1606. 10.1056/NEJMoa1207756 23094721 PMC3532946

[B85] Libby P. (2012). Inflammation in atherosclerosis. Arterioscler. Thromb. Vasc. Biol. 32 (9), 2045–2051. 10.1161/ATVBAHA.108.179705 22895665 PMC3422754

[B86] Linke B. Schreiber Y. Picard-Willems B. Slattery P. Nüsing R. M. Harder S. (2017). Activated platelets induce an anti-inflammatory response of monocytes/macrophages through cross-regulation of PGE2 and cytokines. Mediat. Inflamm. 2017, 1463216. 10.1155/2017/1463216 PMC5448075 28592915

[B87] Luci D. K. Jameson J. B. 2nd Yasgar A. Diaz G. Joshi N. Kantz A. (2014). Synthesis and structure-activity relationship studies of 4-((2-hydroxy-3-methoxybenzyl)amino)benzenesulfonamide derivatives as potent and selective inhibitors of 12-lipoxygenase. J. Med. Chem. 57 (2), 495–506. 10.1021/jm4016476 24393039 PMC3967794

[B88] Mahajan A. Brunson A. Adesina O. Keegan T. H. M. Wun T. (2022). The incidence of cancer-associated thrombosis is increasing over time. Blood Adv. 6, 307–320. 10.1182/bloodadvances.2021005590 34649273 PMC8753193

[B89] Mammadova-Bach E. Gil-Pulido J. Sarukhanyan E. Burkard P. Shityakov S. Schonhart C. (2020). Platelet glycoprotein VI promotes metastasis through interaction with cancer cell-derived galectin-3. Blood 135 (14), 1146–1160. 10.1182/blood.2019002649 32040544

[B90] Mammadova-Bach E. Zigrino P. Brucker C. Bourdon C. Freund M. De Arcangelis A. (2016). Platelet integrin α6β1 controls lung metastasis through direct binding to cancer cell-derived ADAM9. JCI Insight 1, e88245. 10.1172/jci.insight.88245 27699237 PMC5033851

[B91] Mannori G. Crottet P. Cecconi O. Hanasaki K. Aruffo A. Nelson R. M. (1995). Differential colon cancer cell adhesion to E-P-and L-selectin: role of mucin-type glycoproteins. Cancer Res. 55 (19), 4425–4431. 7545541

[B92] Mantovani A. Allavena P. Sica A. Balkwill F. (2008). Cancer-related inflammation. Nature 454 (7203), 436–444. 10.1038/nature07205 18650914

[B93] Mantovani A. Pierotti M. A. (2008). Cancer and inflammation: a complex relationship. Cancer Lett. 267 (2), 180–181. 10.1016/j.canlet.2008.05.003 18562089

[B94] Marrugo-Ramírez J. Mir M. Samitier J. (2018). Blood-based cancer biomarkers in liquid biopsy: a promising non-invasive alternative to tissue biopsy. Int. J. Mol. Sci. 19 (10), 2877. 10.3390/ijms19102877 30248975 PMC6213360

[B95] Martin P. Leibovich S. J. (2005). Inflammatory cells during wound repair: the good, the bad and the ugly. Trends Cell Biol. 15 (11), 599–607. 10.1016/j.tcb.2005.09.002 16202600

[B96] Massagué J. Obenauf A. C. (2016). Metastatic colonization by circulating tumour cells. Nature 529 (7586), 298–306. 10.1038/nature17038 26791720 PMC5029466

[B97] Mayer K. Hein-Rothweiler R. Schüpke S. Janisch M. Bernlochner I. Ndrepepa G. (2021). Efficacy and safety of revacept, a novel lesion-directed competitive antagonist to platelet glycoprotein VI, in patients undergoing elective percutaneous coronary intervention for stable ischemic heart disease: the randomized, double-blind, placebo-controlled ISAR-PLASTER phase 2 trial. JAMA Cardiol. 6 (7), 753–761. 10.1001/jamacardio.2021.0475 33787834 PMC8014195

[B98] Menter D. G. Schilsky R. L. DuBois R. N. (2010). Cyclooxygenase-2 and cancer treatment: understanding the risk should be worth the reward. Clin. Cancer Res. 16 (5), 1384–1390. 10.1158/1078-0432.CCR-09-0788 20179228 PMC4307592

[B99] Michael J. V. Wurtzel J. G. T. Mao G. F. Rao A. K. Kolpakov M. A. Sabri A. (2017). Platelet microparticles infiltrating solid tumors transfer miRNAs that suppress tumor growth. Blood 130 (5), 567–580. 10.1182/blood-2016-11-751099 28500171 PMC5542851

[B100] Mills G. B. Moolenaar W. H. (2003). The emerging role of lysophosphatidic acid in cancer. Nat. Rev. Cancer 3, 582–591. 10.1038/nrc1143 12894246

[B101] Mitrugno A. Williams D. Kerrigan S. W. Moran N. (2014). A novel and essential role for FcγRIIa in cancer cell-induced platelet activation. Blood 123, 249–260. 10.1182/blood-2013-03-492447 24258815

[B102] Momi S. Falcinelli E. Petito E. Ciarrocca Taranta G. Ossoli A. Gresele P. (2022). Matrix metalloproteinase-2 on activated platelets triggers endothelial PAR-1 initiating atherosclerosis. Eur. Heart J. 43 (6), 504–514. 10.1093/eurheartj/ehab631 34529782

[B103] Moser C. L. Gould K. A. McNeley M. K. Shoemaker A. R. Dove W. F. (1995). ApcMin: a mouse model for intestinal and mammary tumorigenesis. Eur. J. Cancer 31A, 1061–1064. 10.1016/0959-8049(95)00181-h 7576992

[B104] Nagalla S. Shaw C. Kong X. Kondkar A. A. Edelstein L. C. Ma L. (2011). Platelet microRNA-mRNA coexpression profiles correlate with platelet reactivity. Blood 117 (19), 5189–5197. 10.1182/blood-2010-09-299719 21415270 PMC3109541

[B105] National Institute for Health and Care (2020). Excellence NICE-effectiveness of aspirin in the prevention of colorectal cancer in people with Lynch syndrome. London, UK: National Institute for Health and Care. 32730002

[B106] Negus R. P. Stamp G. W. Hadley J. Balkwill F. R. (1997). Quantitative assessment of the leukocyte infiltrate in ovarian cancer and its relationship to the expression of C-C chemokines. Am. J. Pathol. 150 (5), 1723–1734. 9137096 PMC1858213

[B107] Nierodzik M. L. Karpatkin S. (2006). Thrombin induces tumor growth, metastasis, and angiogenesis: evidence for a thrombin-regulated dormant tumor phenotype. Cancer Cell 10 (5), 355–362. 10.1016/j.ccr.2006.10.002 17097558

[B108] Nilsson R. J. Balaj L. Hulleman E. van Rijn S. Pegtel D. M. Walraven M. (2011). Blood platelets contain tumor-derived RNA biomarkers. Blood 118 (13), 3680–3683. 10.1182/blood-2011-03-344408 21832279 PMC7224637

[B109] Nilsson R. J. Karachaliou N. Berenguer J. Gimenez-Capitan A. Schellen P. Teixido C. (2016). Rearranged EML4-ALK fusion transcripts sequester in circulating blood platelets and enable blood-based crizotinib response monitoring in non-small-cell lung cancer. Oncotarget 7 (1), 1066–1075. 10.18632/oncotarget.6279 26544515 PMC4808052

[B110] Oshima M. Dinchuk J. E. Kargman S. L. Oshima H. Hancock B. Kwong E. (1996). Suppression of intestinal polyposis in Apc delta716 knockout mice by inhibition of cyclooxygenase 2 (COX-2). Cell 87 (5), 803–809. 10.1016/s0092-8674(00)81988-1 8945508

[B111] Patrignani P. Patrono C. (2015). Cyclooxygenase inhibitors: from pharmacology to clinical read-outs. Biochim. Biophys. Acta 1851, 422–432. 10.1016/j.bbalip.2014.09.016 25263946

[B112] Patrignani P. Patrono C. (2016). Aspirin and cancer. J. Am. Coll. Cardiol. 68, 967–976. 10.1016/j.jacc.2016.05.083 27561771

[B113] Patrignani P. Patrono C. (2018). Aspirin, platelet inhibition and cancer prevention. Platelets 29 (8), 779–785. 10.1080/09537104.2018.1492105 29985750

[B114] Patrignani P. Tacconelli S. Piazuelo E. Di Francesco L. Dovizio M. Sostres C. (2014). Reappraisal of the clinical pharmacology of low-dose aspirin by comparing novel direct and traditional indirect biomarkers of drug action. J. Thromb. Haemost. 12 (8), 1320–1330. 10.1111/jth.12637 24942808

[B165] Patrignani P. Tacconelli S. Contursi A. Piazuelo E. Bruno A. Nobili S. (2024). Optimizing aspirin dose for colorectal cancer patients through deep phenotyping using novel biomarkers of drug action. Front. Pharmacol. 29;15, 1362217. 10.3389/fphar.2024.1362217 PMC10941341 38495101

[B115] Patrono C. Baigent C. (2019). Role of aspirin in primary prevention of cardiovascular disease. Nat. Rev. Cardiol. 16 (11), 675–686. 10.1038/s41569-019-0225-y 31243390

[B116] Patrono C. García Rodríguez L. A. Landolfi R. Baigent C. (2005). Low-dose aspirin for the prevention of atherothrombosis. N. Engl. J. Med. 353 (22), 2373–2383. 10.1056/NEJMra052717 16319386

[B117] Patrono C. Patrignani P. García Rodríguez L. A. (2001). Cyclooxygenase selective inhibition of prostanoid formation: transducing biochemical selectivity into clinical read-outs. J. Clin. Invest 108, 7–13. 10.1172/JCI13418 11435450 PMC209347

[B118] Petito E. Momi S. Gresele P. (2017). “The migration of platelets and their interaction with other migrating cells,” in Platelets in thrombotic and non-thrombotic disorders. Editors Gresele P. Kleiman N. Lopez J. Page C. (Cham, Switzerland: Springer), 337–351. Ray MK, Fagan SP, and Brunicardi.

[B119] Pitchford S. C. Momi S. Baglioni S. Casali L. Giannini S. Rossi R. (2008). Allergen induces the migration of platelets to lung tissue in allergic asthma. Am. J. Respir. Crit. Care Med. 177 (6), 604–612. 10.1164/rccm.200702-214OC 18096710

[B120] Pitchford S. C. Momi S. Giannini S. Casali L. Spina D. Page C. P. (2005). Platelet P-selectin is required for pulmonary eosinophil and lymphocyte recruitment in a murine model of allergic inflammation. Blood 105 (5), 2074–2081. 10.1182/blood-2004-06-2282 15528309

[B121] Pitchford S. C. Yano H. Lever R. Riffo-Vasquez Y. Ciferri S. Rose M. J. (2003). Platelets are essential for leukocyte recruitment in allergic inflammation. J. Allergy Clin. Immunol. 112 (1), 109–118. 10.1067/mai.2003.1514 12847487

[B122] Placke T. Örgel M. Schaller M. Jung G. Rammensee H. G. Kopp H. G. (2012). Platelet-derived MHC class I confers a pseudonormal phenotype to cancer cells that subverts the antitumor reactivity of natural killer immune cells. Cancer Res. 72, 440–448. 10.1158/0008-5472.CAN-11-1872 22127925

[B123] Puhm F. Boilard E. Machlus K. R. (2021). Platelet extracellular vesicles: beyond the blood. Arterioscler. Thromb. Vasc. Biol. 41 (1), 87–96. 10.1161/ATVBAHA.120.314644 33028092 PMC7769913

[B124] Rachidi S. Metelli A. Riesenberg B. Wu B. X. Nelson M. H. Wallace C. (2017). Platelets subvert T cell immunity against cancer via GARP-TGFβ axis. Sci. Immunol. 2 (11), eaai7911. 10.1126/sciimmunol.aai7911 28763790 PMC5539882

[B125] Rana R. Huang T. Koukos G. Fletcher E. K. Turner S. E. Shearer A. (2018). Noncanonical matrix metalloprotease 1–protease-activated receptor 1 signaling drives progression of atherosclerosis. Arterioscler. Thromb. Vasc. Biol. 38, 1368–1380. 10.1161/ATVBAHA.118.310967 29622563 PMC6076425

[B126] Ratajczak J. Wysoczynski M. Hayek F. Janowska-Wieczorek A. Ratajczak M. Z. (2006). Membrane-derived microvesicles: important and underappreciated mediators of cell-to-cell communication. Leukemia 20, 1487–1495. 10.1038/sj.leu.2404296 16791265

[B127] Risitano A. Beaulieu L. M. Vitseva O. Freedman J. E. (2012). Platelets and platelet-like particles mediate intercellular RNA transfer. Blood 119, 6288–6295. 10.1182/blood-2011-12-396440 22596260 PMC3383198

[B128] Rondina M. T. Weyrich A. S. Zimmerman G. A. (2013). Platelets as cellular effectors of inflammation in vascular diseases. Circ. Res. 112 (11), 1506–1519. 10.1161/CIRCRESAHA.113.300512 23704217 PMC3738064

[B129] Ross R. Bowen-Pope D. F. Raines E. W. (1985). Platelets, macrophages, endothelium, and growth factors. Their effects upon cells and their possible roles in atherogenesis. Ann. N. Y. Acad. Sci. 454, 254–260. 10.1111/j.1749-6632.1985.tb11865.x 3907465

[B130] Rovati G. Contursi A. Bruno A. Tacconelli S. Ballerini P. Patrignani P. (2022). Antiplatelet agents affecting GPCR signaling implicated in tumor metastasis. Cells 11 (4), 725. 10.3390/cells11040725 35203374 PMC8870128

[B131] Rumbaut R. E. Thiagarajan P. (2010). Platelet-vessel wall interactions in hemostasis and thrombosis. San. Rafael Morgan Claypool Life Sci. 2, 1–75. 10.4199/C00007ED1V01Y201002ISP004 21452436

[B132] Sacco A. Bruno A. Contursi A. Dovizio M. Tacconelli S. Ricciotti E. (2019). Platelet-specific deletion of cyclooxygenase-1 ameliorates dextran sulfate sodium-induced colitis in mice. J. Pharmacol. Exp. Ther. 370 (3), 416–426. 10.1124/jpet.119.259382 31248980

[B133] Santoso S. Sachs U. J. Kroll H. Linder M. Ruf A. Preissner K. T. (2002). The junctional adhesion molecule 3 (JAM-3) on human platelets is a counterreceptor for the leukocyte integrin Mac-1. J. Exp. Med. 196 (5), 679–691. 10.1084/jem.20020267 12208882 PMC2194005

[B134] Serrano D. Patrignani P. Stigliano V. Turchetti D. Sciallero S. Roviello F. (2022). Aspirin colorectal cancer prevention in Lynch syndrome: recommendations in the era of precision medicine. Genes (Basel) 13 (3), 460. 10.3390/genes13030460 35328014 PMC8952565

[B135] Sim M. M. S. Shiferawe S. Wood J. P. (2023). Novel strategies in antithrombotic therapy: targeting thrombosis while preserving hemostasis. Front. Cardiovasc Med. 10, 1272971. 10.3389/fcvm.2023.1272971 37937289 PMC10626538

[B136] Simon D. I. Chen Z. Xu H. Li C. Q. Dong Jf McIntire L. V. (2000). Platelet glycoprotein ibalpha is a counterreceptor for the leukocyte integrin Mac-1 (CD11b/CD18). J. Exp. Med. 192 (2), 193–204. 10.1084/jem.192.2.193 10899906 PMC2193258

[B137] Sitia G. Aiolfi R. Di Lucia P. Mainetti M. Fiocchi A. Mingozzi F. (2012). Antiplatelet therapy prevents hepatocellular carcinoma and improves survival in a mouse model of chronic hepatitis B. Proc. Natl. Acad. Sci. U. S. A. 109, E2165–E2172. 10.1073/pnas.1209182109 22753481 PMC3420192

[B138] Skog J. Würdinger T. van Rijn S. Meijer D. H. Gainche L. Sena-Esteves M. (2008). Glioblastoma microvesicles transport RNA and proteins that promote tumour growth and provide diagnostic biomarkers. Nat. Cell Biol. 10 (12), 1470–1476. 10.1038/ncb1800 19011622 PMC3423894

[B139] Slatter D. A. Percy C. L. Allen-Redpath K. Gajsiewicz J. M. Brooks N. J. Clayton A. (2018). Enzymatically oxidized phospholipids restore thrombin generation in coagulation factor deficiencies. JCI Insight 3 (6), e98459. 10.1172/jci.insight.98459 29563336 PMC5926910

[B140] Smyth S. S. McEver R. P. Weyrich A. S. Morrell C. N. Hoffman M. R. Arepally G. M. (2009). Platelet functions beyond hemostasis. J. Thromb. Haemost. 7 (11), 1759–1766. 10.1111/j.1538-7836.2009.03586.x 19691483

[B141] Stanger L. Holinstat M. (2023). Bioactive lipid regulation of platelet function, hemostasis, and thrombosis. Pharmacol. Ther. 246, 108420. 10.1016/j.pharmthera.2023.108420 37100208 PMC11143998

[B142] Tang M. Jiang L. Lin Y. Wu X. Wang K. He Q. (2017). Platelet microparticle-mediated transfer of miR-939 to epithelial ovarian cancer cells promotes epithelial to mesenchymal transition. Oncotarget 8, 97464–97475. 10.18632/oncotarget.22136 29228624 PMC5722576

[B143] Thomas C. P. Morgan L. T. Maskrey B. H. Murphy R. C. Kühn H. Hazen S. L. (2010). Phospholipid-esterified eicosanoids are generated in agonist-activated human platelets and enhance tissue factor-dependent thrombin generation. J. Biol. Chem. 285 (10), 6891–6903. 10.1074/jbc.M109.078428 20061396 PMC2844139

[B144] Timár J. Rásó E. Honn K. V. Hagmann W. (1999). 12-lipoxygenase expression in human melanoma cell lines. Adv. Exp. Med. Biol. 469, 617–622. 10.1007/978-1-4615-4793-8_89 10667390

[B145] Tourdot B. E. Holinstat M. (2017). Targeting 12-lipoxygenase as a potential novel antiplatelet therapy. Trends Pharmacol. Sci. 38 (11), 1006–1015. 10.1016/j.tips.2017.08.001 28863985

[B146] Tran D. Q. Andersson J. Wang R. Ramsey H. Unutmaz D. Shevach E. M. (2009). GARP (LRRC32) is essential for the surface expression of latent TGF-beta on platelets and activated FOXP3+ regulatory T cells. Proc. Natl. Acad. Sci. U. S. A. 106 (32), 13445–13450. 10.1073/pnas.0901944106 19651619 PMC2726354

[B147] Tripodi A. Mannucci P. M. (2011). The coagulopathy of chronic liver disease. N. Engl. J. Med. 365, 147–156. 10.1056/NEJMra1011170 21751907

[B148] Turnage R. H. LaNoue J. L. Kadesky K. M. Meng Y. Myers S. I. (1985). Thromboxane A2 mediates increased pulmonary microvascular permeability after intestinal reperfusion. J. Appl. Physiol. 82 (2), 592–598. 10.1152/jappl.1997.82.2.592 9049742

[B149] Tuxhorn J. A. Ayala G. E. Rowley D. R. (2001). Reactive stroma in prostate cancer progression. J. Urol. 166 (6), 2472–2483. 10.1016/s0022-5347(05)65620-0 11696814

[B150] Tuxhorn J. A. Ayala G. E. Smith M. J. Smith V. C. Dang T. D. Rowley D. R. (2002). Reactive stroma in human prostate cancer: induction of myofibroblast phenotype and extracellular matrix remodeling. Clin. Cancer Res. 8 (9), 2912–2923. 12231536

[B151] Ungerer M. Rosport K. Bültmann A. Piechatzek R. Uhland K. Schlieper P. (2011). Novel antiplatelet drug revacept (Dimeric Glycoprotein VI-Fc) specifically and efficiently inhibited collagen-induced platelet aggregation without affecting general hemostasis in humans. Circulation 123, 1891–1899. 10.1161/CIRCULATIONAHA.110.980623 21502572

[B152] Varon D. Shai E. (2015). Platelets and their microparticles as key players in pathophysiological responses. J. Thromb. Haemost. 13 (Suppl. 1), S40–S46. 10.1111/jth.12976 26149049

[B153] Vasina E. M. Cauwenberghs S. Feijge M. A. Heemskerk J. W. Weber C. Koenen R. R. (2011). Microparticles from apoptotic platelets promote resident macrophage differentiation. Cell Death and Dis. 2, e211. 10.1038/cddis.2011.94 PMC3186911 21956548

[B154] Vitiello L. Spoletini I. Gorini S. Pontecorvo L. Ferrari D. Ferraro E. (2014). Microvascular inflammation in atherosclerosis. IJC Metab. Endocr. 3, 1–7. 10.1016/j.ijcme.2014.03.002

[B155] Wang D. DuBois R. N. (2013). An inflammatory mediator, prostaglandin E2, in colorectal cancer. Cancer J. 19 (6), 502–510. 10.1097/PPO.0000000000000003 24270349 PMC4797645

[B156] Weyrich A. S. Elstad M. R. McEver R. P. McIntyre T. M. Moore K. L. Morrissey J. H. (1996). Activated platelets signal chemokine synthesis by human monocytes. J. Clin. Invest 97 (6), 1525–1534. 10.1172/JCI118575 8617886 PMC507213

[B157] Williams J. R. Khandoga A. L. Goyal P. Fells J. I. Perygin D. H. Siess W. (2009). Unique ligand selectivity of the GPR92/LPA5 lysophosphatidate receptor indicates role in human platelet activation. J. Biol. Chem. 284, 17304–17319. 10.1074/jbc.M109.003194 19366702 PMC2719366

[B158] Willis Fox O. Preston R. J. S. (2020). Molecular basis of protease-activated receptor 1 signaling diversity. J. Thromb. Haemost. 18, 6–16. 10.1111/jth.14643 31549766

[B159] Wong C. C. Baum J. Silvestro A. Beste M. T. Bharani-Dharan B. Xu S. (2020). Inhibition of IL-1β by canakinumab may be effective against diverse molecular subtypes of lung cancer: an exploratory analysis of the CANTOS trial. Cancer Res. 80 (24), 5597–5605. 10.1158/0008-5472.CAN-19-3176 33023946

[B160] Yang J. Hirata T. Croce K. Merrill-Skoloff G. Tchernychev B. Williams E. (1999). Targeted gene disruption demonstrates that P-selectin glycoprotein ligand 1 (PSGL-1) is required for P-selectin-mediated but not E-selectin-mediated neutrophil rolling and migration. J. Exp. Med. 190 (12), 1769–1782. 10.1084/jem.190.12.1769 10601352 PMC2195714

[B161] Yang R. Y. Rabinovich G. A. Liu F. T. (2008). Galectins: structure, function and therapeutic potential. Expert Rev. Mol. Med. 10, e17. 10.1017/S1462399408000719 18549522

[B162] Yeung J. Tourdot B. E. Fernandez-Perez P. Vesci J. Ren J. Smyrniotis C. J. (2014). Platelet 12-LOX is essential for FcγRIIa-mediated platelet activation. Blood 124 (14), 2271–2279. 10.1182/blood-2014-05-575878 25100742 PMC4183986

[B163] Yoshimoto T. Arakawa T. Hada T. Yamamoto S. Takahashi E. (1992). Structure and chromosomal localization of human arachidonate 12-lipoxygenase gene. J. Biol. Chem. 267, 24805–24809. 10.1016/s0021-9258(18)35835-6 1447217

[B164] Zhu Y. Zhu M. Lance P. (2012). IL1β-mediated Stromal COX-2 signaling mediates proliferation and invasiveness of colonic epithelial cancer cells. Exp. Cell Res. 318 (19), 2520–2530. 10.1016/j.yexcr.2012.07.021 22884582

